# SLC7A5 correlated with malignancies and immunotherapy response in bladder cancer

**DOI:** 10.1186/s12935-024-03365-7

**Published:** 2024-05-24

**Authors:** Chunyu Zhang, Yanan Wang, Xiangdong Guo, Zhihua Wang, Jiatong Xiao, Zhi Liu

**Affiliations:** 1 Department of Urology, Tongji Hospital, Tongji Medical College, Huazhong University of Science and Technology, Wuhan, China; 2 Departments of Urology, Xiangya Hospital, Central South University, Changsha, China; 3 Department of Urology, The Second Affiliated Hospital of Guizhou Medical University, Guiyang, China

**Keywords:** Bladder cancer, Immunotherapy, SLC7A5, Tumor microenvironment

## Abstract

**Background:**

Metabolic reprogramming contributes to bladder cancer development. This study aimed to understand the role of SLC7A5 in bladder cancer.

**Methods:**

We systematically analyzed the correlation between SLC7A5 and bladder cancer through various approaches, including bioinformatics, western blotting, cell cycle analysis, cell proliferation assays, and invasion experiments. We also investigated the immunological features within the tumor microenvironment (TME), encompassing cancer immune cycles, immune modulators, immune checkpoints, tumor-infiltrating immune cells (TIIC), T cell inflammation scores, and treatment responses. Additionally, for a comprehensive assessment of the expression patterns and immunological roles of SLC7A5, pan-cancer analysis was performed using cancer genomics datasets.

**Results:**

SLC7A5 was associated with adverse prognosis in bladder cancer patients, activating the Wnt pathway and promoting bladder cancer cell cycle progression, proliferation, migration, and invasion. Based on the evidence that SLC7A5 positively correlated with immunomodulators, TIIC, the cancer immune cycle, immune checkpoint and T cell inflammation scores, we also found that SLC7A5 was associated with the inflammatory tumor immune microenvironment. EGFR-targeted therapy, cancer immunotherapy, and radiation therapy were effective for patients with high SLC7A5 expression in bladder cancer. Low SLC7A5 patients were, however, sensitive to targeted therapies and anti-angiogenic therapy, such as blocking β-catenin network, PPAR-γ and FGFR3 signaling. Anti-SLC7A5 combined with cancer immunotherapy may have greater effectiveness than either therapy alone. Furthermore, we observed specific overexpression of SLC7A5 in TME of various cancers.

**Conclusion:**

SLC7A5 can predict therapeutic response to immunotherapy, radiotherapy and chemotherapy in bladder cancer patients. Targeting SLC7A5 in combination with immunotherapy may be a potentially appropriate treatment option.

**Supplementary Information:**

The online version contains supplementary material available at 10.1186/s12935-024-03365-7.

## Introduction

As one of the most common urological malignancies, Bladder cancer (BLCA) caused an estimated 81,180 new cases and 17,100 deaths in 2022 [1]. Depending on whether the cancer is non-muscle-invasive muscle-invasive, or metastatic, the disease can present in various forms. A high mutational load and mutational spectrum characterize muscle-invasive bladder cancer (MIBC), which responds well to treatment [2–4]. Although immunotherapy and neoadjuvant therapies have shown great promise for BLCA patients relative to past therapies, it is important to recognize that response rates still need to be improved [5]. There are two major challenges in this area including the development of robust biomarkers for predicting which patients will respond to specific immune checkpoint inhibitors (ICIs) and the assignment of other patients to new treatments being evaluated in clinical trials, and enhancing ICI through the development of novel combination therapies combined with targeted therapies.

Cancer cells require large amounts of nutrients, including glucose, amino acids and others. The upregulation of nutrient transporter proteins can facilitate meeting their needs. Unlike glucose transporter proteins, amino acid transporter proteins include a transporter protein whose expression is specialized for cancer cells, called solute carrier family 7 member 5 (SLC7A5) [6]. Cancer cells express SLC7A5 primarily in their plasma membranes. Several studies have demonstrated poor patient outcomes associated with high SLC7A5 expression in cancers of various tissues [7–9]. SLC7A5 is essential for cancer cell growth, and studies have demonstrated that pharmacological inhibition and knockdown/knockout of SLC7A5 inhibit the proliferation of cancer cells and xenograft tumors [10–12]. Through downregulation of the mTORC1 signaling pathway, SLC7A5 inhibition suppresses protein synthesis [13]. Based on the importance of SLC7A5, JPH203 (KYT0353) has been developed as one of the highest-affinity inhibitors of SLC7A5, guided by the structure-activity relationship of SLC7A5 ligands, with minimal impact on other transport proteins. In Phase I clinical trials, JPH203 has demonstrated significant anticancer effects [6]. As a result, SLC7A5 may be a molecular target in the diagnosis and treatment of cancer.

SLC7A5 mediates almost entirely leucine uptake in BLCA T24 cell lines [14]. The knockdown of SLC7A5 by siRNA significantly reduced the growth of T24 cells [15]. There is still uncertainty as to the relationship between SLC7A5 and prognosis, tumor immune microenvironment, and therapeutic response in patients with BLCA. Therefore, we have further validated the role of SLC7A5 on BLCA progression and its predictive value for BLCA patients’ prognosis and therapeutic response through bioinformatics analysis of TCGA and GEO cohorts, sequencing analysis of Xiangya cohort, qPCR, CCK8, colony-forming assay, wound healing and transwell experiments.

## Methods

### Preprocessing and retrieval of data

TCGA: From a cancer genome atlas (https://portal.gdc.cancer.gov/), RNA sequencing data (FPKM values) and clinical data was downloaded. FPKM was subsequently converted to TPM values. Four hundred and twelve BLCA samples were included in the study after a genomic and clinicopathological filter was applied.

External BLCA cohort: Four BLCA cohorts (GSE32894, GSE48075, GSE48276, GSE87304) were downloaded from Gene Expression Omnibus (GEO).

Immunotherapy cohort: In IMvigor210 cohort, there are 348 BLCA samples who have been treated with anti-PD-L1 immunotherapy and these samples have been sourced from the following website: www.researchpub.gene.com/imvigor210corebiologies/. GSE135222 and GSE78200 were downloaded from the GEO database. Gide 2019, KIM 2018 and VanAllen 2015 were collected from the TIDE website.

Xianya cohort: A total of 57 BLCA samples and 13 normal tissues collected from our hospital were identified and sequenced. These samples’ RNA sequencing data were analyzed using TPM values. More information is provided in a previously published paper [16].

### Multi-omics analysis

Copy number was analyzed using GISTIC [17]. DNA methylation was analyzed using UALCAN [18]. For protein expression levels, the analysis was performed using CPTAC (https://proteomics.cancer.gov/programs/cptac). For RNA methylation molecular correlation analysis, Sangerbox was used for analysis (http://sangerbox.com/home.html). The m6a methylation site was predicted using SRAMP [19]. Protein interactions were predicted using the String database [20].

### Cell culture

All cells were grown in a cell incubator at 37 °C. With the exception of SW780 cells, all other cells are cultured in a 5% CO_2_ environment. 10% fetal bovine serum (FBS, Gibco, NY, USA), 100 units /ml streptomycin and 100 mg/ml penicillin (Gibco, NY, USA) were added to all cell culture-medium. Bladder cancer cell lines UMUC3, SW780 and RT4 were cultured in DMEM (Gibco, NY, USA), while 5637, RT112 and T24 were cultured in 1640 medium (Gibco, NY, USA). Normal bladder epithelial cells SVHUC were cultured in F12K (Gibco, NY, USA).

### Cell transfection and gene silencing, overexpression

We obtained a short hairpin RNA (shRNA) targeting SLC7A5 from Sango Biotech (Shanghai, China). Sango Biotech (Shanghai, China) synthesized the pWPI lentiviral vector used for the SLC7A5 overexpression construct. As directed by the manufacturer (Invitrogen, Carlsbad, USA), transfection was carried out using Lipofectamine 2000. Cancer cells were incubated with medium containing the virus for approximately 72 hours after transfection. The cells were then used for further testing after another 72 hours. shSLC7A5-1: 5’-ACATTGTGCTGGCATTATA-3’; shSLC7A5-2: 5’- TGACCAACCTGGCCTACTT-3’; shSLC7A5-3: 5’- GCATCGGCTTCACCATCAT-3’.

### RNA isolation, reverse transcription and real-time PCR

Total RNA was isolated in RNase-free water (AGbio, Hunan, China) using TRIzol Reagent (Invitrogen, Carlsbad, CA, USA) from tissues and cells. The PrimeScript RT Reagent Kit was used to reverse transcribe total RNA into complementary DNA (AGbio, Hunan, China). SYBR Green PCR Reagent (AGbio, Hunan, China) was used to performed Real-time fluorescent quantitative PCR. Experiments were performed according to manufacturer instructions, and each reaction was repeated three times. The qRT-PCR primers for SLC7A5 were as follows: forward: 5′- GATGACGCTGCTCTACGCCTTC − 3′ and reverse: 5′- CTGAGGATGATGGTGAAGCCGATG − 3′. The qRT-PCR primers for GAPDH were as follows: forward: 5′- CAAGGCTGTGGGCAAGGTCATC − 3′ and reverse: 5′- GTGTCGCTGTTGAAGTCAGAGGAG − 3′.

### Western blot

Extract protein from cells. Load protein samples onto polyacrylamide gel and separate proteins by electrophoresis. Transfer separated proteins onto PVDF membrane. Block nonspecific binding sites with skim milk powder. Incubate membrane with specific primary antibody to bind target protein. Wash membrane with buffer to remove unbound primary antibody. Incubate membrane with specific secondary antibody. Wash membrane with buffer to remove unbound secondary antibody. Visualize target protein using a chemiluminescent substrate.

### CCK-8 assay

At a density of 2 × 103 cells/well, cells were inoculated into 96-well plates. 10 µl Cell Counting Kit-8 (CCK-8) reagent (NCM Biotech, Suzhou, China) was added to each well. An absorbance measurement at 450 nm was conducted using a microplate reader (Thermo, USA) after 2 h of incubation with 5% CO2 at 37 °C. Each reaction was repeated three times.

### Colony-forming assay

Colony formation assay was used to observe its effect on the survival of cell colonies. Cells were inoculated at 1 × 10^3^ cells/well in 6-well plates until colonies were visible (about 14 days). The cells were fixed using4% paraformaldehyde and crystalline violet staining solution was used to stain them.

### Wound-healing

Cells were inoculated in 6-well plates and cultured until they reached 90% confluence. Scratch with a sterile 200 µL pipette tip. Photographs were taken at 0 and 24 h after scratching.

### Transwell assays

100 µl of cells in serum-free medium were placed in the upper chamber of transwell inserts (Corning, USA) for the migration assay. 100 µl of cells in serum-free medium were placed in the upper chamber of transwell inserts with matrigel (Corning, USA) for the invasion assay. A medium supplemented with 10% FBS (600 uL) was added to the lower chamber and cultured for 12–48 h at 37 °C in 5% CO2. As cells migrate or invade the membrane, paraformaldehyde is used to fix them, crystal violet is used to stain them, and cotton swab are used to remove the surviving cells in the upper chamber membrane. Imaging and counting. The experiment was repeated three times independently.

### Evaluation of the immunological characteristics of the TME in BLCA

As part of the anticancer immune response in BLCA TME, the tumor cells release cancer cell antigens (Step 1); The antigens produced by tumor cells are presented to immune cells (e.g. dendritic cells) in the TME (Step 2); Antigen-presenting cells (APCs) carry the antigens and initiate and activate the immune response inside the TME (Step 3); After initiating the immune system, chemokines and cytokines are released, leading to the recruitment of immune cells into the TME ( Steps 4–5); The TME contains cytotoxic immune cells, such as CD8 ^+^ T cells and natural killer T cells, which recognize cancer cells (Step 6) and kill them (Step 7) [21]. The fate of tumor cells was determined by the strength of the seven steps. In addition, we calculated the levels of TIICs by employing several independent algorithms based on RNA-seq data, including TIMER, TISIDB, CIBERSORT-ABS, quanTIseq, TIP, xCell and MCP-counter [22–27]. A T-cell inflammation score (TIS), which predicts clinical response to immune checkpoint blockade (ICB), can reflect pre-existing anticancer immunity in TME [28]. Using ssGSEA, we calculated enrichment scores for several immunotherapeutic response pathways. Finally, we screened and collected 20 suppressive immune checkpoints, including PD-1, PD-L1, LAG3, TIGIT and CTLA-4. Our previous studies have well described these immunological features [29].

### BLCA treatment options and clinical sensitivity prediction

On the basis of Genomics of Drug Sensitibity in Cancer (GDSC) (https://www.cancerrxgene.org/) data, we assessed response to six common chemotherapeutic agents in patients with terminal BLCA using the pRRophetic software package. High-risk scoring groups and low-risk scoring groups were compared on the 50% inhibitory concentration (IC50) of six chemotherapeutic agents.

### Animal experiment

6-8-week-old C57 mice were anesthetized, and then 30 µl of MB49 cells were injected into the bladder wall using a syringe. The tumor development was observed using live animal imaging. At the experimental endpoint, bladder tumors were harvested and processed into single-cell suspensions for flow cytometry analysis.

### Statistical analysis

Visualization and statistical analysis of data were carried out using R software (version 4.0.5) and Graphpad Prism 9. Using Pearson or Spearman correlation analysis, we determined the relationship between continuous variables. The t-tests were used for continuous variables that fitted a normal distribution between binary groups, while Fisher’s exact probability and Student’s t-tests were used for comparisons between groups. The correlation between risk scores and prognosis was assessed using multivariate and univariate Cox regression analyses. Otherwise, the Mann Whitney U test was used. Data are expressed as SD ± mean. The experiments were performed three times and the P values were calculated two-sidedly. *P < 0.05 were considered statistically significant, while **P < 0.01 and ***P<0.001 were considered highly significant.

## Results

### Multi-omic analysis of SLC7A5 in BLCA

**Fig. 1:**
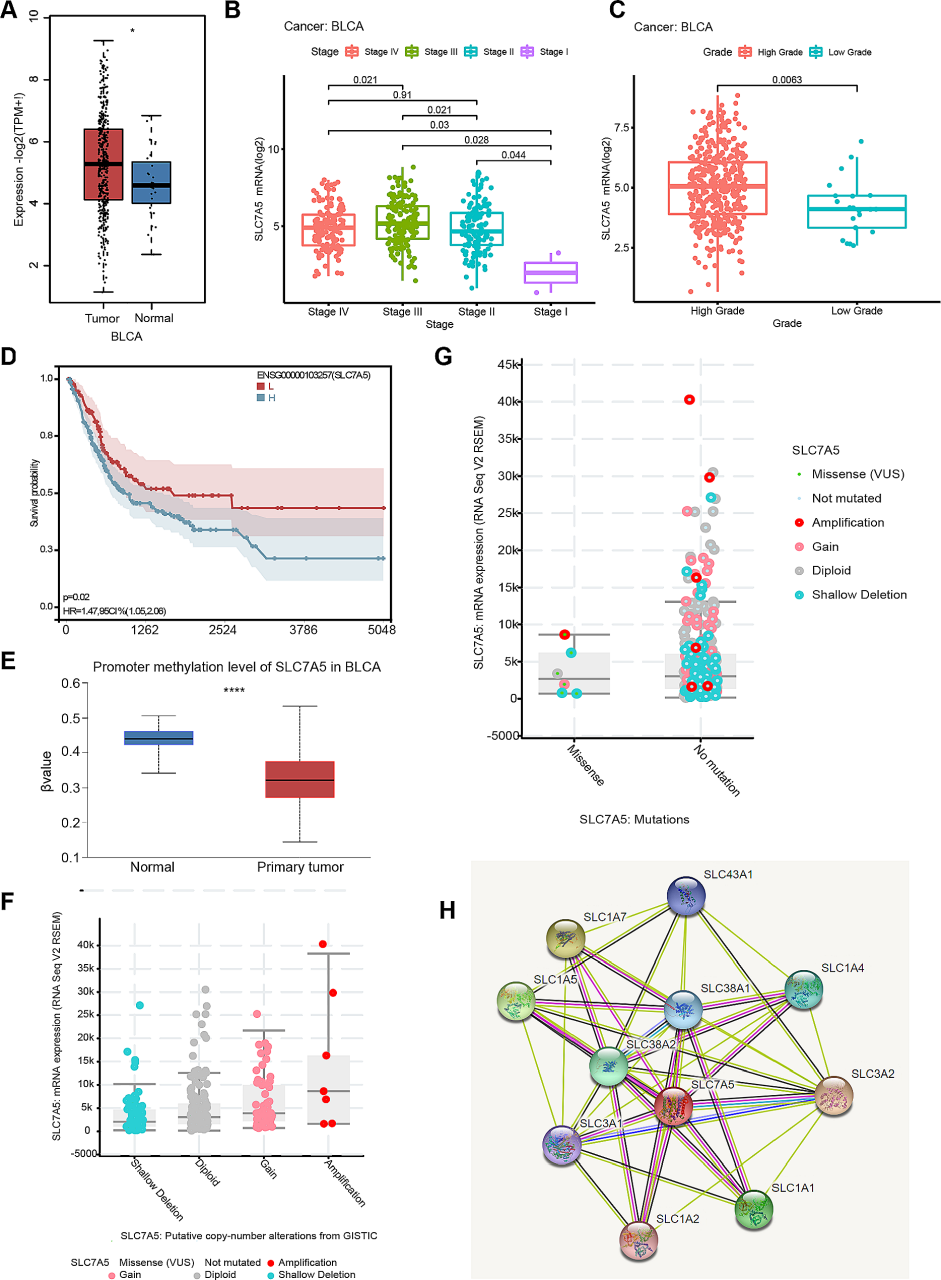
Multi-omic analysis of SLC7A5 in BLCA. **A**. The mRNA expression of SLC7A5 in TCGA-BLCA. **B**. On the basis of the TCGA data, the expression level of the SLC7A5 gene was evaluated according to the main pathological stages of BLCA (stages I, II, III, and IV). **C**. A grade-specific analysis of the expression levels of the SLC7A5 gene was conducted using TCGA data. **D**. We performed overall survival analyses of BLCA in TCGA by SLC7A5 gene expression. **E**. We performed a correlation analysis between SLC7A5 expression and promoter methylation level of TCGA in BLCA. **F**. The GISTIC database was analyzed for correlation of putative copy-number alteration with the expression of SLC7A5. **G**. The GISTIC database was analyzed for correlation of mutations with the expression of SLC7A5. **H.** The STRING tool enabled us to obtain the SLC7A5-binding proteins that have been experimentally determined. *P < 0.05; **P < 0.01; ***P < 0.001

It has been shown that SLC7A5 was significantly overexpressed in a variety of tumors and was associated with poor prognosis [8]. In the TCGA database analysis, SLC7A5 expression was significantly associated with higher stage and grade in BLCA compared to normal tissue. (Fig. 1A-C) The survival analysis showed that BLCA patients with high expression of SLC7A5 had a poorer prognosis. (Fig. 1D) A DNA methylation mark is often referred to as a silencing epigenetic mark that is tightly linked to gene expression [30]. In the UALCAN database, SLC7A5 DNA promoter region methylation was significantly reduced in BLCA, which is consistent with the trend of upregulation of SLC7A5 expression. (Fig. 1E) In addition, the GISTIC database showed that SLC7A5 expression was closely associated with CNV, in which amplification was more common. (Fig. 1F-G, Supplement Fig. 1) At the proteomic level, SLC7A5 is mainly closely related to SLC family members, and there is a potential for interaction, such as SLC1A2, SLC1A1, SLC38A1, and so on. (Fig. 1H)

### SLC7A5 promotes the proliferation, invasion and migration of BLCA

**Fig. 2:**
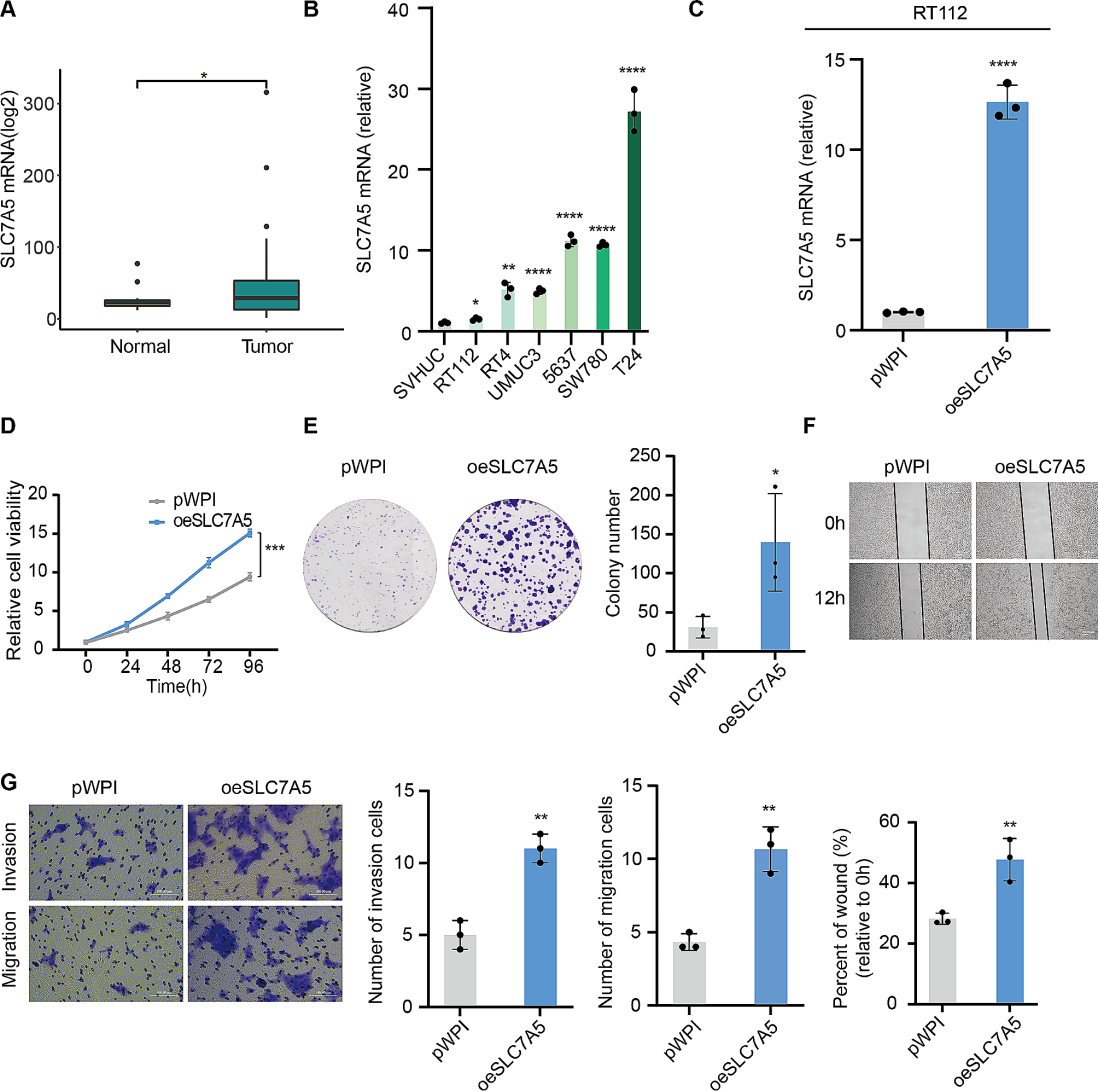
SLC7A5 overexpression promoted the proliferation, invasion and migration of bladder cancer cells. **A**. The mRNA expression of SLC7A5 in Xiangya cohort. **B**. qPCR analysis of the SLC7A5 mRNA level in SVHUC, RT112, RT4, UMUC3, 5637, SW780 and T24. **C**. qPCR analysis of the SLC7A5 mRNA level in RT112 cell lines respectively transfected with oeSLC7A5 and control pWPI. **D**. Viability of RT112-transfected with oeSLC7A5 versus control pWPI as determined by the CCK8 assay. **E**. The proliferation of RT112 cells transfected with oeSLC7A5 or control pWPI was evaluated by colony formation assay. **F**. The migration of RT112 cells transfected with oeSLC7A5 and control pWPI were tested for wound healing assays. **G**. Migration and invasion of RT112 cells transfected with oeSLC7A5 and control pWPI in transwell assays. *P < 0.05; **P < 0.01; ***P < 0.001

**Fig. 3:**
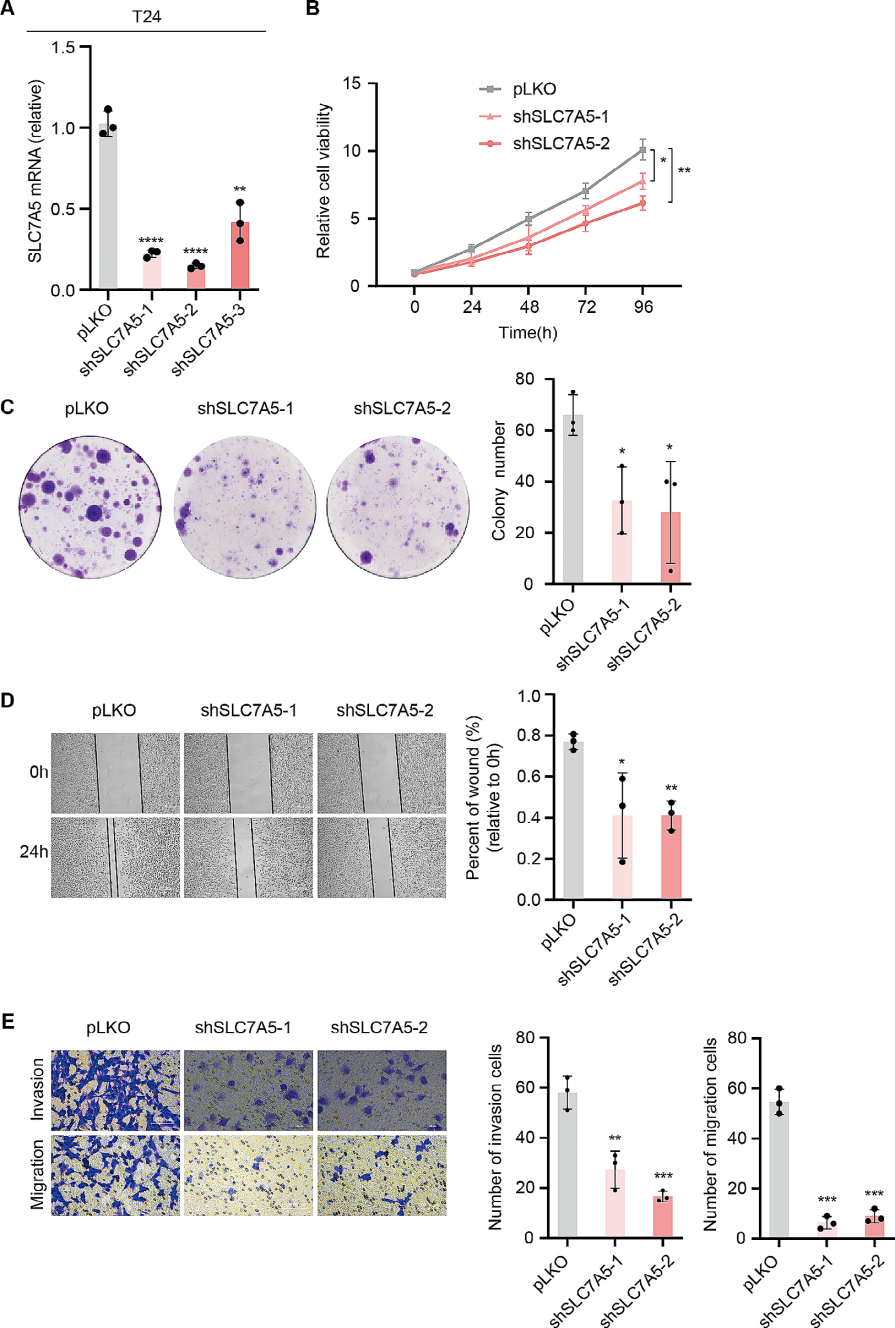
SLC7A5 knockdown inhibited the proliferation, invasion and migration of bladder cancer cells. **A**. qPCR analysis of the SLC7A5 mRNA level in RT112 cell lines respectively transfected with shSLC7A5-1, shSLC7A5-2, shSLC7A5-3 and control pLKO. **B**. Using the CCK8 assay, comparing shSLC7A5-1 and shSLC7A5-2 transfections with control pLKO-transfected T24 cells. **C**. Proliferation assays for T24 cell lines transfected with shSLC7A5-2, shSLC7A5-1, and control pLKO in colony formation assay. **D**. The wound healing assay was performed with T24 cells transfected with shSLC7A5-2, shSLC7A5-1, and control pLKO for the migration **E**. The transwell assay was carried out on T24 cells transfected with shSLC7A5-2, shSLC7A5-1, and pLKO control for migration and invasion. *P < 0.05; **P < 0.01; ***P < 0.001

To further validate the role of SLC7A5 in bladder carcinogenesis and development, we collected BLCA and paracancerous tissues from Xiangya Hospital and sequenced them to establish the Xiangya cohort. BLCA tissues expressed more SLC7A5 than normal tissues according to transcriptome sequencing results. (Fig. 2A) Based on quantitative PCR analysis, SLC7A5 mRNA was significantly upregulated in six BLCA cell lines than normal bladder epithelial cells. (Fig. 2B) Knockdown and overexpression of SLC7A5 in T24 and RT112 cells, respectively, and validation of their transfection efficiency. (Figures 2C and 3A, Supplement Fig. 2) BLCA cells were tested for proliferation by colony formation and CCK8 assays, The results showed that knocking down SLC7A5 significantly reduced the proliferation ability of BLCA cells, while the overexpression was the opposite. (Figures 2D-E and 3B-C) BLCA cells showed a significant decrease in invasion and migration ability after SLC7A5 knockdown and an increase after overexpression of the protein in wound healing and transwell assays. (Figures 2F-G and 3D-E)

### SLC7A5 regulates the Wnt signaling pathway and cell cycle in bladder cancer

**Fig. 4:**
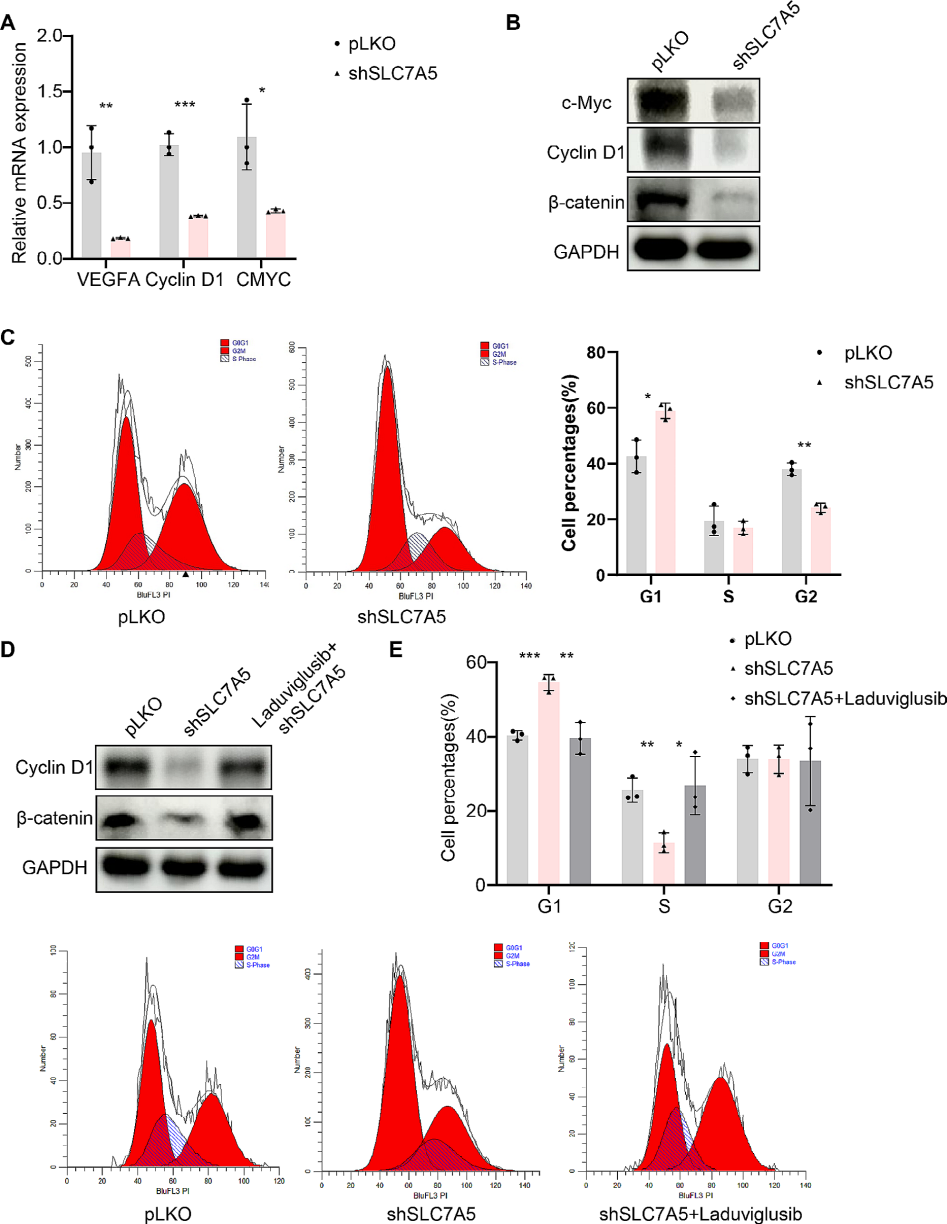
SLC7A5 regulates the Wnt signaling pathway and cell cycle in bladder cancer. **A**. qPCR analysis of the VEGFA、Cyclin D1 and CMYC mRNA level in RT112 cell lines respectively transfected with shSLC7A5 and control pLKO. **B**. A. WB analysis of the β-catenin、Cyclin D1 and c-Myc expression level in RT112 cell lines respectively transfected with shSLC7A5 and control pLKO. **C**. Flow cytometry analysis was performed to assess the cell cycle in pLKO and shSLC7A5 groups. **D**. After the treatment of Laduviglusib, WB analysis of the β-catenin and Cyclin D1 expression level. **E**. After the treatment of Laduviglusib, flow cytometry analysis was performed to assess the cell cycle. *P < 0.05; **P < 0.01; ***P < 0.001

TCGA-BLCA data were stratified into two groups based on SLC7A5 expression levels, and a KEGG enrichment analysis was conducted. The results revealed significant enrichment in immune regulation-related pathways such as neutrophil degranulation, neutrophil activation involved in immune response, and antigen processing and presentation of exogenous peptide antigen via MHC class I. (Supplementary Table 1) Additionally, KEGG analysis indicated a close association between SLC7A5, the Wnt pathway, and the cell cycle. (Supplementary Table 1) Western blot (WB) and quantitative polymerase chain reaction (QPCR) results demonstrated a significant reduction in β-catenin expression after SLC7A5 knockdown, accompanied by decreased levels of VEGFA, c-Myc, and Cyclin D1. (Fig. 4A, B) Flow cytometry results indicated an increase in the number of cells in the G0/G1 phase and a decrease in the number of cells in the S phase following SLC7A5 knockdown, suggesting that SLC7A5 knockdown induces cell cycle arrest at the G0/G1 phase in bladder cancer cells. (Fig. 4C) Furthermore, upon treatment with the Wnt pathway activator Laduviglusib, it was observed that the effects on Wnt pathway activation and cell cycle induced by SLC7A5 knockdown were partially restored. (Fig. 4D-E) The regulation of Wnt/β-catenin by SLC7A5 may be due to the action of leucine. SLC7A5 promoted cellular uptake of leucine, which activated Wnt/β-catenin [31].

### SLC7A5 predicts the immune features of BLCA

**Fig. 5:**
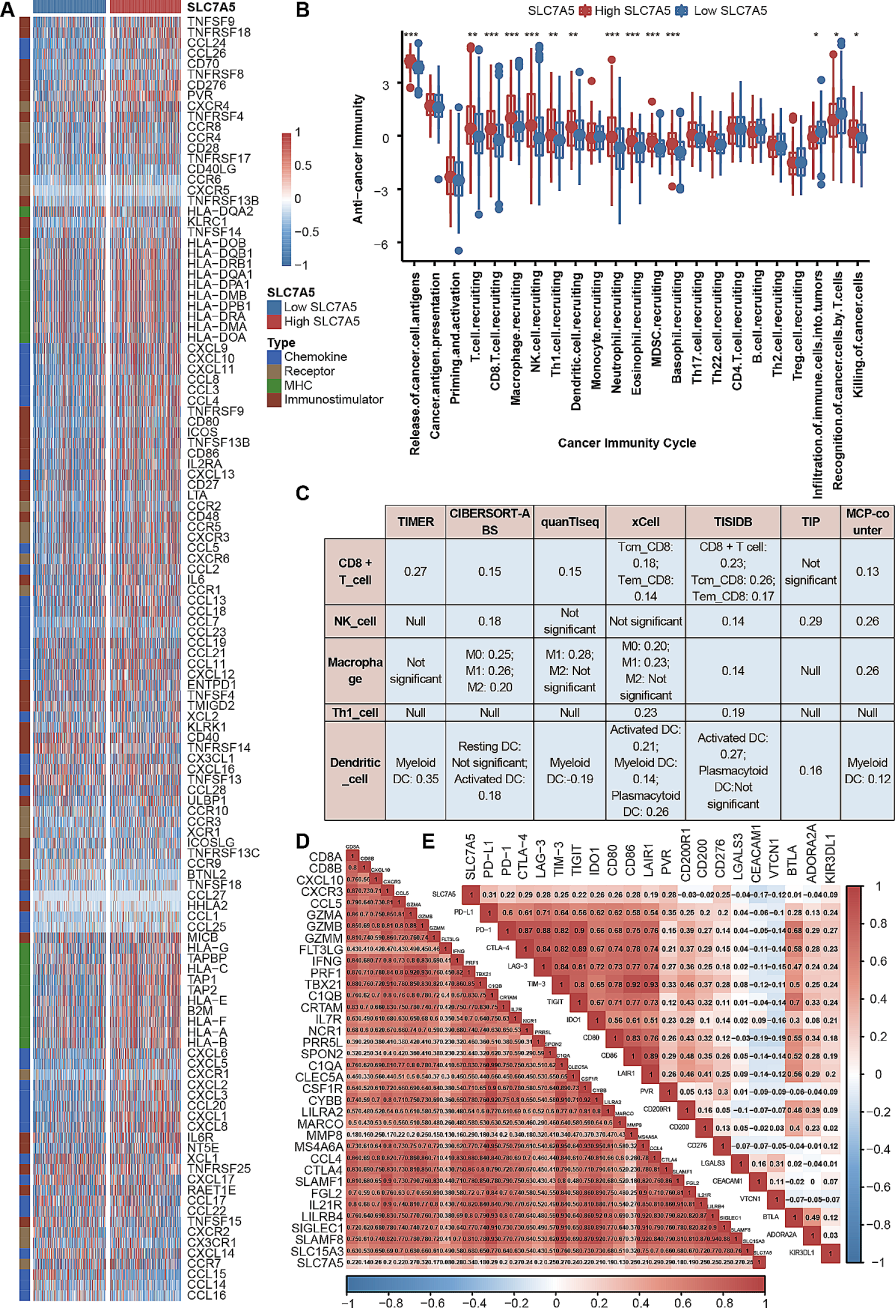
SLC7A5 predicts the immune features of BLCA. **A**. Comparing the expression of 122 immunomodulatory molecules (chemokines, receptors, MHC, and immunostimulators) between BLCA groups with high and low SLC7A5. **B**. There are differences between groups with high- and low-SLC7A5 levels in various stages of the cancer immunity cycle. **C**. Five types of TIICs were calculated using seven independent algorithms, including CD8 ^+^ T cells, NK cells, macrophages, Th1 cells, and dendritic cells, and the correlation was found between SLC7A5 and their infiltration levels. **D**. Effector genes of the above tumor-associated immune cells differ between those with high SLC7A5 levels and those with low SLC7A5. **E**. Correlation between SLC7A5 and 20 inhibitory immune checkpoints. Asterisks indicate a statistically significant p-value calculated using Mann-Whitney U test. The color and values indicate Spearman correlation coefficients. *P < 0.05; **P < 0.01; ***P < 0.001

The emergence of immunotherapy has brought light to patients with advanced BLCA, but the efficacy of immunotherapy or neoadjuvant therapy depends greatly on the tumor immune microenvironment [32]. It can be seen from Fig. 5A that SLC7A5 is positively correlated with most immunomodulators. Most MHC molecules are upregulated in the high SLC7A5 group, indicating that the antigen-presenting ability was upregulated in the high SLC7A5 group. And the key chemokines that induce CD8^ +^ T cell recruitment (CXCL9, CXCL10, CCR3) and the related chemokines and paired receptors (CCL2, CCL3, CCL4, CCL8, CXCL11, CCR1, CCR2, CCR6, CXCR4 etc.) that induce effector TIIC recruitment were positively correlated with SLC7A5.

**Fig. 6:**
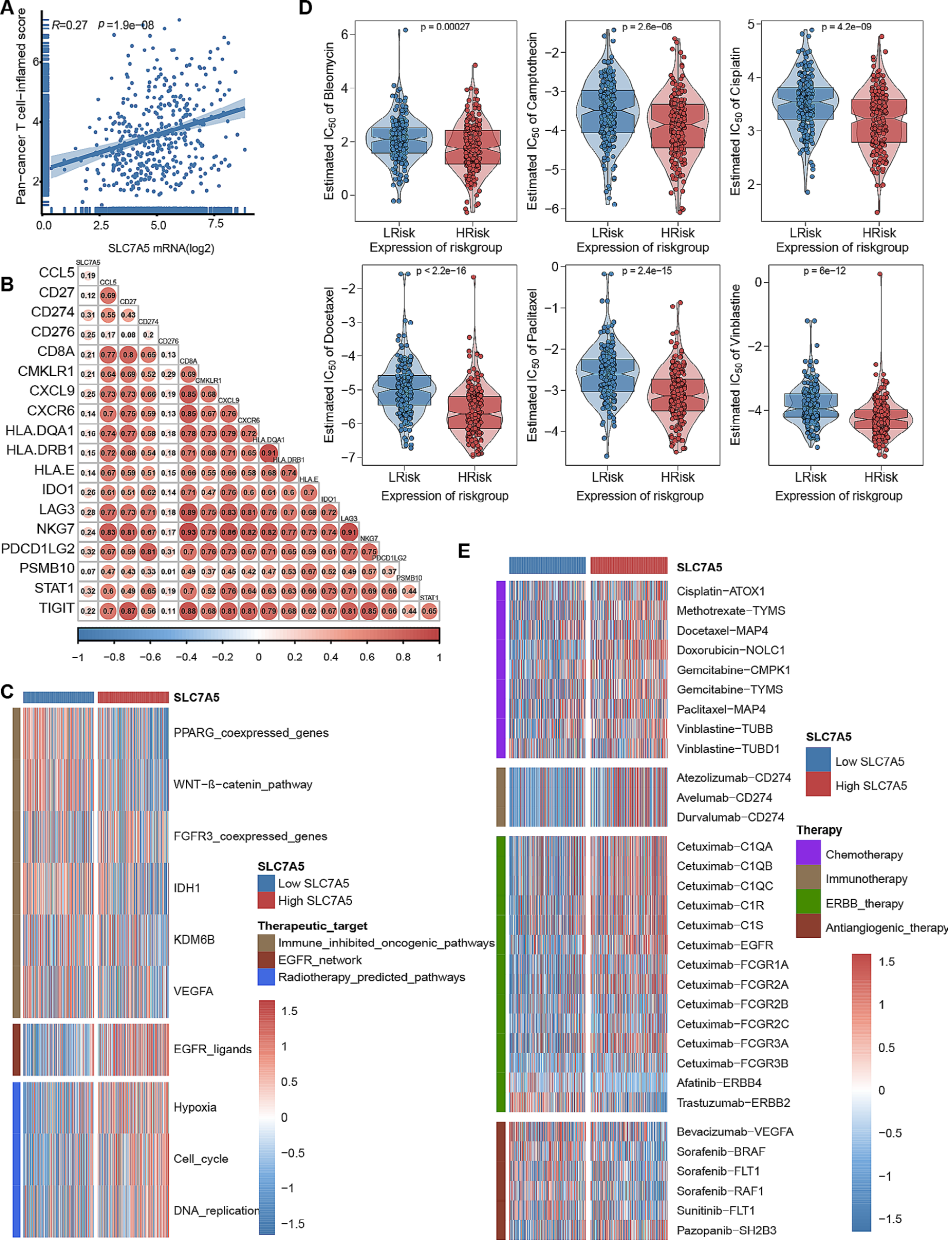
In patients with BLCA, SLC7A5 predicts the therapeutic response to several therapies. **A**-**B**. SLC7A5 and the pan-cancer T cell inflamed score and the individual genes included in the T cell inflamed signature are correlated. It has been shown that a positive correlation exists between the T cell inflamed score and clinical response to cancer immunotherapy. **C**. The correlation between SLC7A5 and the enrichment scores of several therapeutic signatures, including targeted therapy and radiation therapy. **D**. An analysis of the effects of six chemotherapy drugs on high- and low-SLC7A5 groups. **E**. A correlation between SLC7A5 and the BLCA-related drug-target genes screened from Drugbank

Most cancer immune cycles were more active in patients with high SLC7A5. For example, the release of cancer cell antigens (Step 1), the transport of immune cells to the tumor (Step 4) (CD8 T cell recruitment, macrophage recruitment, Th1 cell recruitment, MDSC recruitment, NK cell recruitment, DC recruitment) and the killing of cancer cells (Step 7). (Fig. 5B) Meanwhile, Increased activity of these immune cycles leads to higher levels of TIIC infiltration in BLCA TME (including CD8 T cells, NK cells, Th1 cells, macrophages, and DCs). A cross-validation was performed on seven independent algorithms. (Fig. 5C, supplement Figs. 3, 4, 5, 6, 7, 8 and 9) These results suggest that the high SLC7A5 group is more likely to be an inflammatory phenotype and more sensitive to ICB. In addition, these effector genes of TIIC as well as most immune checkpoints have significant and positive correlations with SLC7A5, including PD-L1, CTLA4, PD-1, LAG3, and TIGIT. (Fig. 5D-E) Further analysis of the relationship between SLC7A5 and TIS revealed that they were positively correlated. (Fig. 6A-B)

Next, we explored the value of SLC7A5 in predicting treatment response to several treatment options. It is possible that patients with high SLC7A5 are more susceptible to EGFR-targeted therapy and radiotherapy, in contrast, low SLC7A5 levels, however, were significantly associated with several immunosuppressive oncogenic pathways, including the Wnt-β-catenin network, IDH1, PPARG network, KDM6B and VEGFA. (Fig. 6C) It may be possible to target these oncogenic pathways to provide therapeutic benefit for patients with BLCA and low SLC7A5. Additionally, chemotherapy drugs were more sensitive to patients with high SLC7A5 levels including cisplatin, paclitaxel, camptothecin, bleomycin, docetaxel and vincristine, as well as immunologic drugs. Additionally, chemotherapy drugs were more sensitive to patients with high SLC7A5 levels (Fig. 6D-E) Based on the results presented here, SLC7A5 may be a potential predictor of the efficacy of ICB in BLCA and may be an indicator of the effectiveness of treatment for BLCA.

### Validation of SLC7A5 as a predictor of immunophenotyping and ICB clinical response in the BLCA immunotherapy cohort (IMvigor210)

**Fig. 7:**
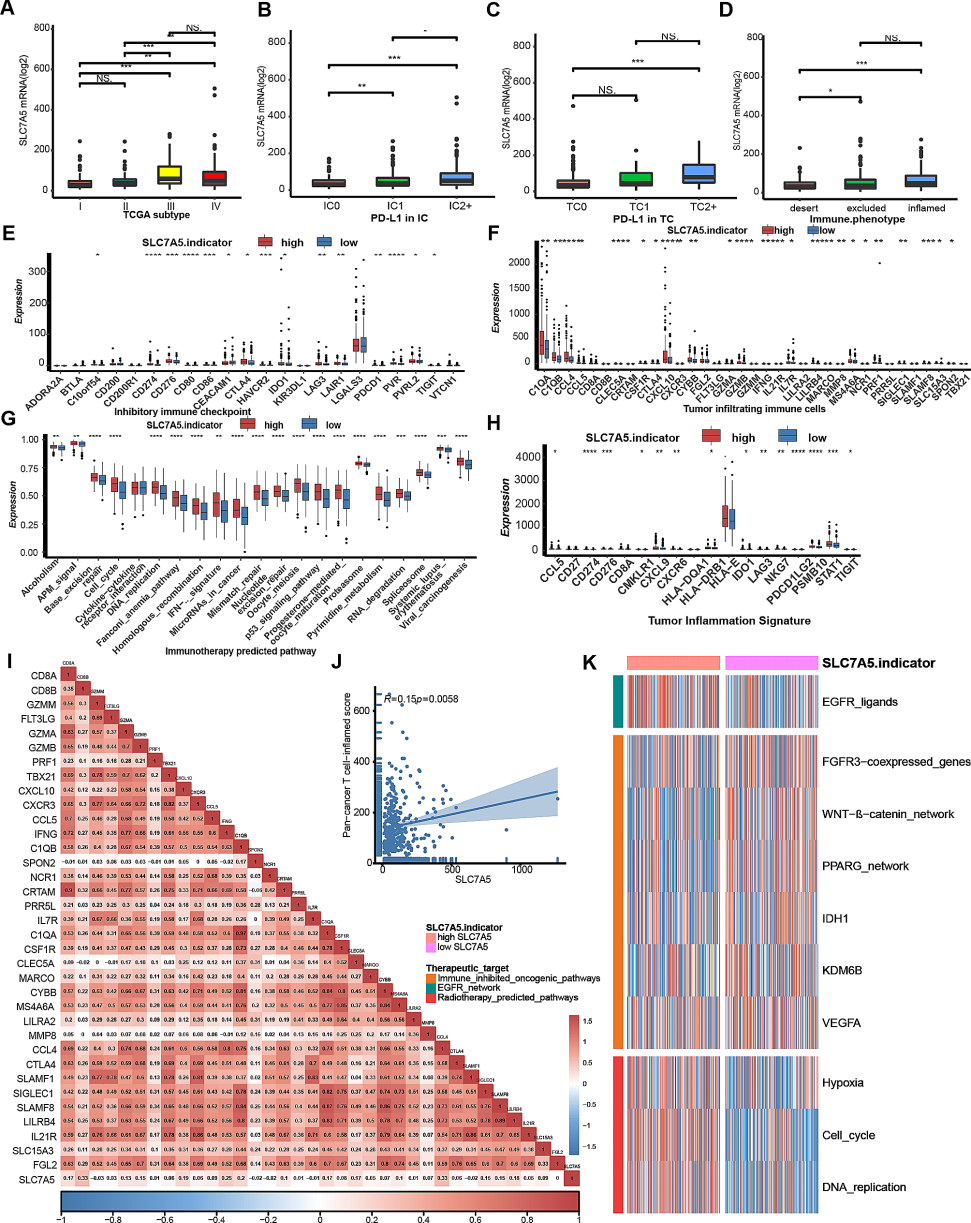
SLC7A5 predicts immunophenotyping and therapeutic response in IMvigor210. **A**. A TCGA subtype (I, II, III, or IV) of BLCA was used to analyze the expression levels of the SLC7A5 gene. **B**. PD-L1 expression levels on immune cells (IC0, IC1 and IC2 + subgroups) of BLCA were analyzed to determine the expression levels of the SLC7A5 gene. **C**. The expression levels of the SLC7A5 gene were analyzed by the PD-L1 expression on tumor cells (TC0, TC1 and TC2 + subgroups) of BLCA. **D**. The expression levels of the SLC7A5 gene were analyzed by the immune phenotype (desert, excluded, inflamed) of BLCA. **E**-**H**. Correlation between SLC7A5 and inhibitory immune checkpoints, effector genes of tumor associated immune cells, enrichment scores of immunotherapies predicted signatures and tumor inflammation signature in BLCA. **I**. Correlation between SLC7A5 and immunomodulators in BLCA. **J**. Correlation between SLC7A5 and pan-cancer T cell inflamed score in BLCA. **K**. The correlation between SLC7A5 and the enrichment scores of several therapeutic signatures, including targeted therapy and radiation therapy. Asterisks indicate statistically significant p values calculated by the Mann-Whitney U test. *P < 0.05; **P < 0.01; ***P < 0.001

In the BLCA immunotherapy cohort IMvigor210, SLC7A5 was significantly associated with the staging of the tumor. (Fig. 7A) Based on the amount of PD-L1 expressed by immune cells (IC0, IC1 and IC2 + subgroups) or tumor cells (TC0, TC1 and TC2 + subgroups) and the level of CD8 T cell infiltration in TME (desert, excluded and inflamed subgroups), patients were divided into subgroups. A lower level of SLC7A5 was found in the IC0 (immune cells with the lowest expression of PD-L1) and TC0 (tumor cells with the lowest expression of PD-L1) subgroups, as well as in the desert phenotype. (Fig. 7B-D) In addition, SLC7A5 was positively correlated with TIS and most immune checkpoints, such as PD-L1, CTLA-4, PD-1, TIGIT and LAG3. (Fig. 7E and J) The high SLC7A5 group also showed significant upregulation of several anti-cancer TIIC effector genes. (Fig. 7F) Several immunotherapy positive gene features, as well as tumor immune features, were positively correlated with SLC7A5. (Fig. 7G-I) These results are all consistent with TCGA results, confirming that the high SLC7A5 group represents an inflammatory phenotype.

**Fig. 8:**
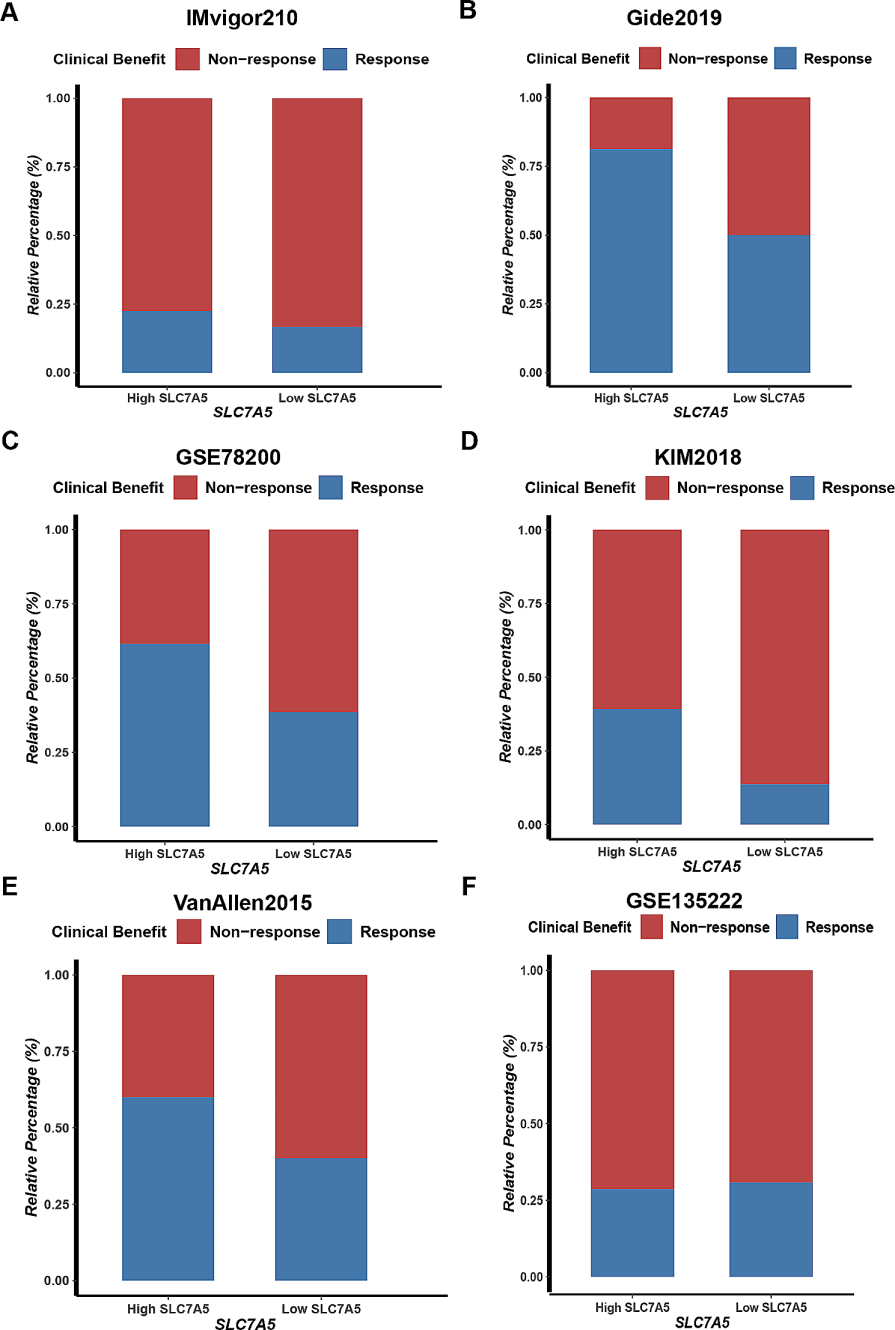
SLC7A5 predicts the response to immunotherapy in several immunotherapy cohorts. A comparison of the proportion of patients who responded to immunotherapy in the low- and high-SLC7A5 groups of the IMvigor210 study, Gide2019, GSE78200, KIM2018, VanAllen2015 and GSE135222

Oncogenic pathways associated with immunosuppression were significantly enriched in the low SLC7A5 group of the IMvigor210 cohort such as Wnt-β-catenin network, PPARG network, and IDH1, while EGFR-targeted therapy and radiotherapy were more beneficial for BLCA patients with high SLC7A5. (Fig. 7K) Furthermore, the analysis of the relationship between efficacy response and SLC7A5 expression in the IMvigor210, Gide2019, GSE78200, KIM2018, VanAllen2015 and GSE135222 immunotherapy cohorts showed that a high SLC7A5 expression was associated with a higher immunotherapy response rate. (Fig. 8) This evidence again confirms that SLC7A5 is a significant predictor of cancer immunotherapy response.

### Verification of the tumor immunological role of SLC7A5 through Xiangya cohort, several external cohorts, and in vivo and in vitro experiments

**Fig. 9:**
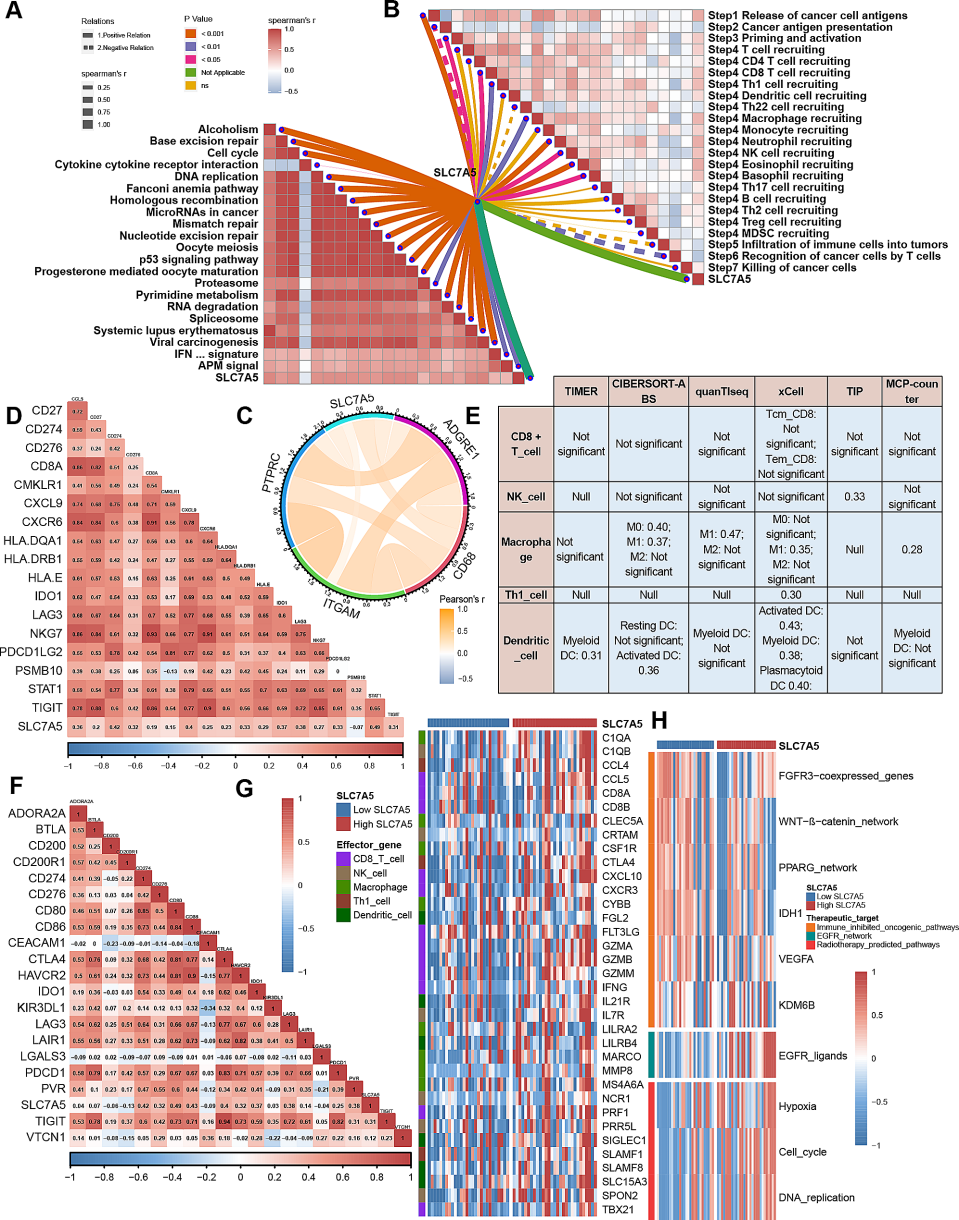
Roles of SLC7A5 in predicting immune phenotypes and therapeutic response in the Xiangya cohort. **A**. Correlations between SLC7A5 and the enrichment scores of immunotherapy-predicted pathways. **B**. Correlations between SLC7A5 and the steps of the cancer immunity cycle. **C**. Correlations between SLC7A5 and four critical marker genes of macrophages. **D**. Correlations between SLC7A5 and the T cell inflamed score. **E**. Correlations between SLC7A5 and the infiltration levels of five tumor-associated immune cells (CD8^ +^ T cells, NK cells, macrophages, Th1 cells, and dendritic cells). **F**. Correlations between SLC7A5 and immune checkpoints. **G**. A correlation between SLC7A5 and tumor-associated immune cells effector genes. **H**. The correlation between SLC7A5 and the enrichment scores of several therapeutic signatures, including targeted therapy and radiation therapy. Solid lines represent positive correlations, dashed lines represent negative correlations. The thickness of the line indicates the strength of the correlation

A positive correlation was found between SLC7A5 and many anti-cancer immune steps in our own cohort (Xiangya cohort). (Fig. 9B) A positive correlation was also found between SLC7A5 and the levels of CD8 T cells, NK cells, Th1 cells, DC cells and macrophages during six independent algorithms. (Fig. 9E) Also, SLC7A5 was positively correlated with four critical marker genes of macrophages. (Fig. 9C) Positive correlations were found between SLC7A5 and features associated with immune checkpoints and ICB response. (Fig. 9A, D, F and G) According to these findings, SLC7A5 can effectively stratify the immunophenotypes of BLCAs. SLC7A5 was also capable of predicting clinical response to other therapies, including radiotherapy, EGFR-targeted therapy and several therapies targeting immunosuppressive oncogenic pathways. (Fig. 9H) Moreover, we performed validation in four other external cohorts of BLCA (GSE32894, GSE48075, GSE48276, GSE87304) and obtained the same results. (Supplement Figs. 10–13) Coculture of bladder cancer cell lines overexpressing SLC7A5 with human peripheral blood CD8^+^ T cells revealed that overexpression of SLC7A5 enhances the cytotoxicity of CD8^+^ T cells. (Supplement Fig. 14) In order to further simulate the in vivo environment, we constructed a bladder transplantation tumor model and found that overexpression of SLC7A5 significantly inhibited tumor growth. This contradicted the previous results showing that overexpression of SLC7A5 promoted tumor proliferation, invasion, and migration. Therefore, we examined the infiltration and function of CD8^+^ T cells in bladder tumors. The results showed that after overexpression of SLC7A5, the infiltration of CD8^+^ T cells significantly increased, and their cytotoxic activity also significantly increased. This may be because the impact of SLC7A5 on the tumor immune microenvironment is greater than its impact on the tumor itself, thus exerting an anti-cancer effect. (Supplement Fig. 15)

### Pan-cancer analysis of clinical and immunological characteristics and immunotherapeutic response of SLC7A5

**Fig. 10:**
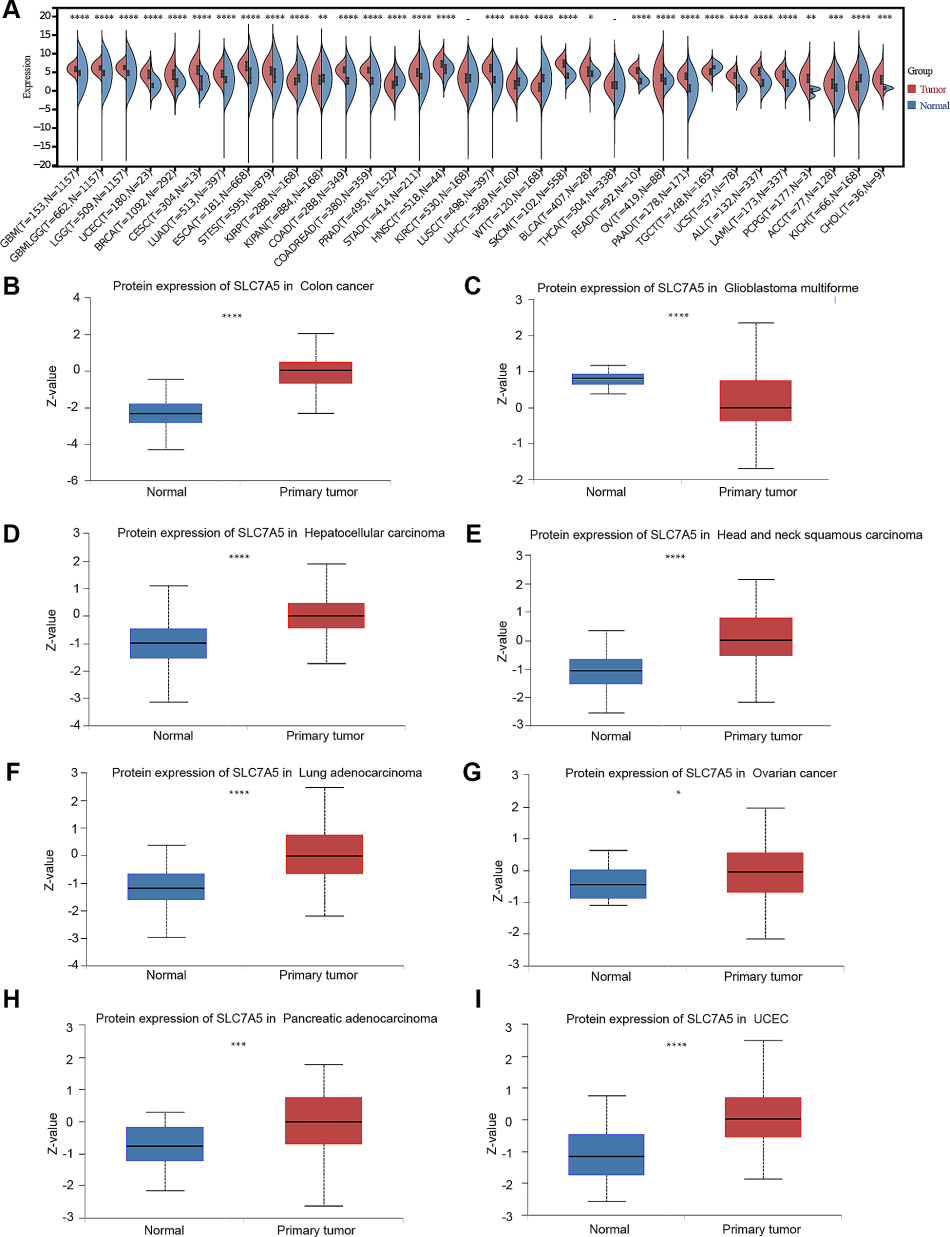
Expression pattern of SLC7A5 in pan-cancers. **A**. TCGA combined with GTEx mRNA expression patterns for SLC7A5 of pan-cancers. **B**-**I**. The protein expression pattern of SLC7A5 of pan-cancers in CPTAC. *P < 0.05; **P < 0.01; ***P < 0.001

We further evaluated the role of SLC7A5 in other cancers. The TCGA database expression data revealed that SLC7A5 is highly expressed in most cancers (such as UCEC, BRCA, CHOL, and others) compared to normal tissues after a comprehensive analysis. (Fig. 10A) CPTAC database analysis showed that SLC7A5 protein was highly expressed in colon cancer, hepatocellular carcinoma, head and neck squamous carcinoma, lung adenocarcinoma, ovarian cancer and UCEC. (Fig. 10B-I) The promoter methylation of SLC7A5 showed an opposite trend to the gene expression in most cancers. (Supplement Fig. 16) This further suggests that SLC7A5 expression may be influenced by DNA methylation. A strong positive correlation was also observed between the expression of SLC7A5 and the expression of m6A, m5C, and m1A molecules. And SRAMP predicted that m6A modification sites on SLC7A5 mRNA were highly abundant. (Supplement Figs. 17 and 18) Pan-cancer analysis showed that SLC7A5 was significantly associated with the stage and grade of multiple tumors. In KIRC and LGG, the higher the malignancy of tumor, the higher the expression of SLC7A5. (Supplement Fig. 19) In most cancers such as TGCT, LUAD, KIRP, ACC, and BRCA, the higher the tumor stage, the higher the SLC7A5 expression. (Supplement Fig. 20) A pan-cancer survival analysis was performed using Kaplan-Meier analysis, Cox regression model and log-rank test concerning, progression-free survival, overall survival and cancer-specific survival. (Supplement Figs. 21, 22 and 23) As expected, SLC7A5 is expected to be a prognostic biomarker in various cancers, although its prognostic values are variable across cancers. These results, however, need further evaluation, especially using multivariate analysis.

**Fig. 11:**
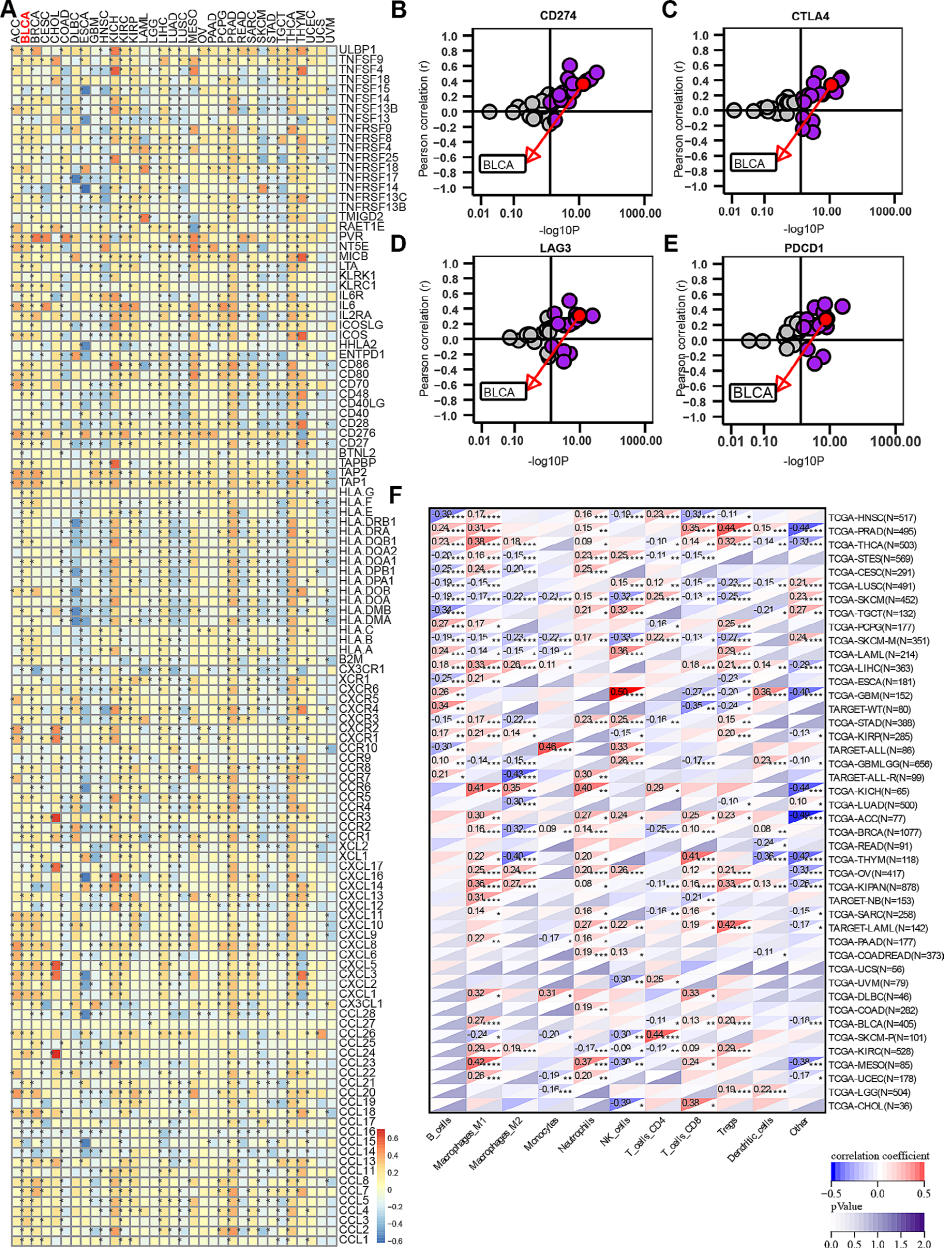
The effect of SLC7A5 on immunological status in pan-cancers. **A**. A correlation exists between SLC7A5 and 122 immunomodulators (chemokines, receptors, MHC, and immunostimulators). **B**-**E**. A correlation is found between SLC7A5 and the immune checkpoints PD-L1, CTLA-4, PD-1, and LAG-3. The dots represent cancer types. The Y-axis represents the Pearson correlation, while the X-axis represents -log10P. **F**. The ssGSEA algorithm was used to calculate the correlation between SLC7A5 and tumor-associated immune cells. Spearman correlation analysis calculates the p-value when the correlation coefficient is significant. Asterisks indicate statistically significant correlation coefficients. *P < 0.05; **P < 0.01; ***P < 0.001

In order to identify cancer types that may benefit from anti-SLC7A5 immunotherapy, a pan-cancer analysis describing SLC7A5’s immune role is essential. The study found a positive correlation between SLC7A5 and immunomodulators in most cancers. (Fig. 11A) The level of TIIC infiltration in TME was estimated using multiple algorithms. SLC7A5 was found to be associated with TIIC in most cancers. (Fig. 11F and Supplement Figs. 24, 25, 26, 27, 28 and 29) Furthermore, we demonstrated a positive correlation between SLC7A5 expression and several immune checkpoints, including PD-L1, CTLA-4, PD-1 and LAG-3. (Fig. 11B-E) Tumor mutational load and microsatellite instability were strongly associated with immunotherapeutic response. Here, in many cancers, we found that SLC7A5 was associated with TMB and MSI. (Supplement Fig. 30) In addition, SLC7A5 is significantly associated with stemness index in many cancers such as CHOL, THYM, and BLCA. (Supplement Fig. 31) In a pan-cancer analysis, SLC7A5 reflects several biological characteristics of TME, such as antitumor immunity, immunogenicity and cancer stemness, which is expected to be a predictor of ICB treatment response and tumor prognosis.

## Discussion

It has been the first-line treatment for NMIBC for decades to combine local tumor resection with intravesical BCG immunotherapy, but the overall outcome has not been satisfactory. Recurrence and progression of disease continue to occur in a significant proportion of patients [5, 33]. Likewise, in the treatment of patients with MIBC and advanced BCa, cisplatin-based chemotherapy coupled with radical cystectomy is unsatisfactory, and due to disease-related comorbidities, some MIBC patients are not eligible for neoadjuvant chemotherapy [34]. These deficiencies are being addressed through the use of immune checkpoint inhibitors. A number of PD-L1 inhibitors and PD-1 inhibitors have been approved for treating platinum-resistant metastatic uroepithelial cancer and MIBC, but only 13–25% objective remission rates have been reported [35]. Therefore, there is an urgent need to develop new therapeutic targets or combination treatment strategies for BLCA patients.

Organic and inorganic solutes are selectively transported across membranes and intracellular organelles by transporter proteins. Nutrients like glucose, amino acids, lipids, vitamins, and minerals are absorbed by them through the epithelium [36]. Growth and proliferation of rapidly growing cancer cells require a large supply of glucose, amino acids, and other nutrients. Upregulation of each nutrient transporter protein contributes to meeting the demand. To meet the increased glucose demand, cancer cells express the Na/glucose cotransport proteins SGLT1 (SLC5A1) and SGLT2 (SLC5A2) [37]. The amino acid transporter proteins, in contrast to glucose transporters, contain a transporter protein whose expression is highly specific for cancer [38]. Our study found that SLC7A5 was significantly upregulated in BLCA tissues and BLCA cell lines, both in the TCGA and Xiangya cohorts.

mTORC1 controls cancer cell growth by activating SLC7A5, which provides leucine, an essential amino acid for cancer cells. In colon cancer, as a result of the strong driving force of Myc oncogene products, SLC7A5 was upregulated, which resulted in metabolic reprogramming associated with cancer development [39]. In view of SLC7A5’s cancer specificity, it has been suggested as a potential molecular target in cancer treatment and diagnosis. To validate SLC7A5 as a molecular target, it is critical to understand the level of SLC7A5 contribution to cellular amino acid uptake. In T24 BLCA cells, leucine uptake is almost exclusively mediated by SLC7A5 [14, 40]. Similarly, in other cancer cell lines, SLC7A5 plays an important role in leucine uptake. It has been demonstrated that SLC7A5 expression is cancer-specific both by immunohistochemistry and PET utilizing SLC7A5-specific probes [41]. SLC7A5-specific PET probe FAMT has been shown to be cancer-specific, distinguishing cancer from non-cancerous lesions, including inflammation [42]. Thus, SLC7A5-specific PET probes have the potential to be developed to solve the problem of conventional FDG-PET concomitant use. SLC7A5-specific PET probes can be used not only as cancer diagnostics for false-positive results, but also as concomitant diagnostics for SLC7A5-targeted therapies to select patients with expected therapeutic benefit. Our study shows that promoting or inhibiting SLC7A5 expression enhances or inhibits the invasion, proliferation and migration of BLCA cells.

It is currently possible to develop antitumor drugs that target SLC7A5 with high affinity One of these compounds, JPH203 (KYT0353), was designed to mimic the conformational design of SLC7A5 ligands. It has the highest binding affinity of all the transporter inhibitors [15, 43]. In preclinical studies, it inhibited tumor growth in vivo without causing significant toxicity. Clinical trials with JPH203 in phase I showed promising results [44]. Since the mechanism of action of SLC7A5 inhibitors is novel and different from other antitumor agents, further synergistic effects can be expected. A number of antitumor drugs, including 5-FU, gemcitabine, cisplatin, and gefitinib, have been reported to have synergistic effects with SLC7A5 inhibitors (or knockdowns/knockouts) [6, 45–47]. JPH203 has recently been shown to significantly reduce cell proliferation when combined with kinase inhibitors that inhibit cell cycle associated proteins [48]. Additionally, the combination reduces the side effects of the antitumor drugs by reducing their doses. In our study, SLC7A5 was found to predict drug therapy response in BLCA, and It is possible that patients with higher SLC7A5 levels might respond better to EGFR-targeted treatment, radiotherapy, and chemotherapy drugs such as cisplatin, paclitaxel, bleomycin, docetaxel and vincristine. In contrast, patients with low SLC7A5 may be more sensitive to drugs targeting immunosuppressive oncogenic pathways such as Wnt-β-catenin protein network, IDH1, PPARG network, KDM6B and VEGFA, and anti-angiogenic drugs.

Moreover, it was shown that SLC7A5 is involved in T cell-mediated immunity. As the heavy chain of the 4F2 antigen, 4F2hc forms a heterodimer with SLC7A5 and is activated by lymphocytes [49, 50]. Based on the molecular characterization of SLC7A5, it has been confirmed that lymphocyte activation also upregulates SLC7A5. It has been shown that NF-kB and AP-1 were involved in the induction of SLC7A5 expression in activated primary human T cells. Inhibition of SLC7A5 resulted in decreased cytokine production through suppression of NFAT and NF-kB activity [51]. SLC7A5 knockout T cells lack clonal amplification, effector differentiation, and can’t reprogram their metabolic response to antigen [52]. A key component of T-cell activation is the uptake of leucine, which activates the expression of mTORC1 and c-Myc. There was a high expression of SLC7A5 in CD4 T cells in atopic dermatitis skin with T-cell activation [53, 54]. In this paper, by analyzing the relationship between SLC7A5 and cancer immune cycle, immunomodulators, TIIC, TIIC effectors and immune checkpoints, it was shown that SLC7A5 was significantly associated with inflammatory immune microenvironment and the patients with high SLC7A5 were more sensitive to immunotherapy. Wang et al.‘s study demonstrated that knocking down SLC7A5 significantly inhibited the proliferation, migration, and invasion of human and mouse TNBC cells [55]. Consistent with our findings, we found that overexpression of SLC7A5 significantly increased the proliferation, invasion, and migration of bladder cancer cells. However, Wang et al. further investigated the role of SLC7A5 in tumor immunity and showed that downregulation of SLC7A5 increased the infiltration of CD8 ^+^ T cells. The combined application of JPH203-mediated SLC7A5 blockade and anti-PD-1 antibody significantly increased the infiltration rate of immune cells and inhibited tumor progression [55]. This contradicts our results, as our study showed that high expression of SLC7A5 in bladder cancer promotes the infiltration and cytotoxic function of CD8 ^+^ T cells in vitro and in vivo, thereby inhibiting tumor growth. This may be due to the different roles of leucine. SLC7A5 is a transporter protein that facilitates the efflux of intracellular glutamine and promotes leucine uptake. Analysis of pan-cancer single-cell sequencing transcriptomics has shown that leucine metabolism can activate H3K27ac modification, promoting T cell responses. Results from various tumor models have demonstrated that leucine intake can modulate tumor immunity and enhance the efficacy of anti-PD-1 therapy. This is consistent with our findings [56].

## Conclusions

As a result, we found that SLC7A5 was associated with the inflammatory tumor microenvironment. Furthermore, SLC7A5 was associated with proliferation, invasion and migration of BLCA, which may not only accurately predict the clinical response of ICB, but also may be a new target for BLCA diagnosis and treatment.

### Electronic supplementary material

Below is the link to the electronic supplementary material.

**Supplementary Material 1: Supplement figure 1**. The GISTIC database was analyzed for putative copy-number alteration, fusions and mutations. Green represents Yes. Gray represents No

**Supplementary Material 2: Supplement figure 2**. Western blot was validated the overexpression and knockdown efficiency of SLC7A5

**Supplementary Material 3: Supplement figure 3**. Based on the CIBERSORT-ABSA algorithm, SLC7A5 and tumor associated immune cells were correlated. Using spearman correlation analysis, the p value was calculated

**Supplementary Material 4: Supplement figure 4**. Based on the MCPCOUNTER algorithm, SLC7A5 and tumor associated immune cells were correlated. Using spearman correlation analysis, the p value was calculated

**Supplementary Material 5: Supplement figure 5**. Based on the QUANTISEQ algorithm, SLC7A5 and tumor associated immune cells were correlated. Using spearman correlation analysis, the p value was calculated.

**Supplementary Material 6: Supplement figure 6**. Based on the TIMER algorithm, SLC7A5 and tumor associated immune cells were correlated. Using spearman correlation analysis, the p value was calculated.

**Supplementary Material 7: Supplement figure 7**. Based on the TIP algorithm, SLC7A5 and tumor associated immune cells were correlated. Using spearman correlation analysis, the p value was calculated.

**Supplementary Material 8: Supplement figure 8**. Based on the TISIDB algorithm, SLC7A5 and tumor associated immune cells were correlated. Using spearman correlation analysis, the p value was calculated

**Supplementary Material 9: Supplement figure 9**. Based on the XCELL algorithm, SLC7A5 and tumor associated immune cells were correlated. Using spearman correlation analysis, the p value was calculated

**Supplementary Material 10: Supplement figure 10**. In GSE32894, SLC7A5 plays a role in predicting immune phenotypes and therapeutic responses. A-D. Correlation between SLC7A5 and inhibitory immune checkpoints, enrichment scores of immunotherapy and effector genes of tumor associated immune cells predicted signatures and tumor inflammation signature in BLCA. E. The correlation between SLC7A5 and the enrichment scores of several therapeutic signatures, including targeted therapy and radiation therapy. Asterisks indicate statistically significant p values calculated by the Mann-Whitney U test. *P＜*0.05*; **P＜*0.01*; ***P＜*0.001*

**Supplementary Material 11: Supplement figure 11**. In GSE48075, SLC7A5 plays a role in predicting immune phenotypes and therapeutic responses. A-D. Correlation between SLC7A5 and inhibitory immune checkpoints, enrichment scores of immunotherapy and effector genes of tumor associated immune cells predicted signatures and tumor inflammation signature in BLCA. E. The correlation between SLC7A5 and the enrichment scores of several therapeutic signatures, including targeted therapy and radiation therapy. Asterisks indicate statistically significant p values calculated by the Mann-Whitney U test. *P＜*0.05*; **P＜*0.01*; ***P＜*0.001*

**Supplementary Material 12: Supplement figure 12**. In GSE48276, SLC7A5 plays a role in predicting immune phenotypes and therapeutic responses. A-D. Correlation between SLC7A5 and inhibitory immune checkpoints, enrichment scores of immunotherapy and effector genes of tumor associated immune cells predicted signatures and tumor inflammation signature in BLCA. E. The correlation between SLC7A5 and the enrichment scores of several therapeutic signatures, including targeted therapy and radiation therapy. Asterisks indicate statistically significant p values calculated by the Mann-Whitney U test. *P＜*0.05*; **P＜*0.01*; ***P＜*0.001*

**Supplementary Material 13: Supplement figure 13**. In GSE87304, SLC7A5 plays a role in predicting immune phenotypes and therapeutic responses. A-D. Correlation between SLC7A5 and inhibitory immune checkpoints, enrichment scores of immunotherapy and effector genes of tumor associated immune cells predicted signatures and tumor inflammation signature in BLCA. E. The correlation between SLC7A5 and the enrichment scores of several therapeutic signatures, including targeted therapy and radiation therapy. Asterisks indicate statistically significant p values calculated by the Mann-Whitney U test. *P＜*0.05*; **P＜*0.01*; ***P＜*0.001*

**Supplementary Material 14: Supplement figure 14**. Results of coculture experiment between T cells and tumor cells. *P＜*0.05*; **P＜*0.01*; ***P＜*0.001*

**Supplementary Material 15: Supplement figure 15**. In vivo animal experiments validate the tumor immunological role of SLC7A5. *P＜*0.05*; **P＜*0.01*; ***P＜*0.001*

**Supplementary Material 16: Supplement figure 16**. Promoter methylation level of SLC7A5 in pan-cancers. *P＜*0.05*; **P＜*0.01*; ***P＜*0.001*

**Supplementary Material 17: Supplement figure 17**. Correlation between SLC7A5 and m1A, m5C and m6A related genes

**Supplementary Material 18: Supplement figure 18**. SRAMP was used to predicts the m6a sites of SLC7A5

**Supplementary Material 19: Supplement figure 19**. In pan-cancers, TCGA data was used to analyze the expression of the SLC7A5 gene based on grade (high grade, low grade).

**Supplementary Material 20: Supplement figure 20**. In pan-cancers, TCGA data was used to analyze the expression of the SLC7A5 gene based on stage (stage I, stage II, stage III, and stage IV)

**Supplementary Material 21: Supplement figure 21**. Prognostic analysis of SLC7A5 for overall survival in pan-cancers. A. Using a univariate Cox regression model, the prognostic significance of SLC7A5 in pan-cancers was assessed. Hazard ratio >1 represented a risk factor, and hazard ratio

**Supplementary Material 22: Supplement figure 22**. Prognostic analysis of SLC7A5 for progression free survival in pan-cancers. A. Using a univariate Cox regression model, the prognostic significance of SLC7A5 in pan-cancers was assessed. Hazard ratio >1 represented a risk factor, and hazard ratio

**Supplementary Material 23: Supplement figure 23**. Prognostic analysis of SLC7A5 for disease specific survival in pan-cancers. A. Using a univariate Cox regression model, the prognostic significance of SLC7A5 in pan-cancers was assessed. Hazard ratio >1 represented a risk factor, and hazard ratio

**Supplementary Material 24: Supplement figure 24**. The CIBERSORT algorithm was used to calculate a correlation between SLC7A5 and tumor-associated immune cells. Correlation coefficient is indicated by the color. When an asterisk appears, it indicates a statistically significant p-value based on spearman correlation analysis. *P＜*0.05*; **P＜*0.01*; ***P＜*0.001*

**Supplementary Material 25: Supplement figure 25**. The EPIC algorithm was used to calculate a correlation between SLC7A5 and tumor-associated immune cells. Correlation coefficient is indicated by the color. When an asterisk appears, it indicates a statistically significant p-value based on spearman correlation analysis. *P＜*0.05*; **P＜*0.01*; ***P＜*0.001*

**Supplementary Material 26: Supplement figure 26**. The MCPcounter algorithm was used to calculate a correlation between SLC7A5 and tumor-associated immune cells. Correlation coefficient is indicated by the color. When an asterisk appears, it indicates a statistically significant p-value based on spearman correlation analysis. *P＜*0.05*; **P＜*0.01*; ***P＜*0.001*

**Supplementary Material 27: Supplement figure 27**. The IPS algorithm was used to calculate a correlation between SLC7A5 and tumor-associated immune cells. Correlation coefficient is indicated by the color. When an asterisk appears, it indicates a statistically significant p-value based on spearman correlation analysis. *P＜*0.05*; **P＜*0.01*; ***P＜*0.001*

**Supplementary Material 28: Supplement figure 28**. The TIMER algorithm was used to calculate a correlation between SLC7A5 and tumor-associated immune cells. Correlation coefficient is indicated by the color. When an asterisk appears, it indicates a statistically significant p-value based on spearman correlation analysis. *P＜*0.05*; **P＜*0.01*; ***P＜*0.001*

**Supplementary Material 29: Supplement figure 29**. The xCELL algorithm was used to calculate a correlation between SLC7A5 and tumor-associated immune cells. Correlation coefficient is indicated by the color. When an asterisk appears, it indicates a statistically significant p-value based on spearman correlation analysis. *P＜*0.05*; **P＜*0.01*; ***P＜*0.001*

**Supplementary Material 30: Supplement figure 30**. In pan-cancers, SLC7A5 is correlated with TMB and MSI. A. Correlation between SLC7A5 and TMB in pan-cancers. B. SLC7A5 and MSI correlation in pan-cancers. Asterisks indicate significant statistical p values calculated using spearman correlation. *P＜*0.05*; **P＜*0.01*; ***P＜*0.001*

**Supplementary Material 31: Supplement figure 31**. Correlations between SLC7A5 and cancer stemness indices across cancers

**Supplementary Material 32:** Supplementary table 1. KEGG analysis of SLC7A5 in TCGA-BLCA. Supplementary table 2. Detailed information of immunotherapy predicted signatures, bladder cancer signatures and other therapeutic signatures

## Data Availability

The datasets used and/or analyzed during the current study are available from the corresponding author on reasonable request.
