# Supplementary figures and images for: SLC7A5 correlated with malignancies and immunotherapy response in bladder cancer
Source: Cancer Cell Int. 2024 May 24;24:182. doi: 10.1186/s12935-024-03365-7 (PMC11127462; doi:10.1186/s12935-024-03365-7)

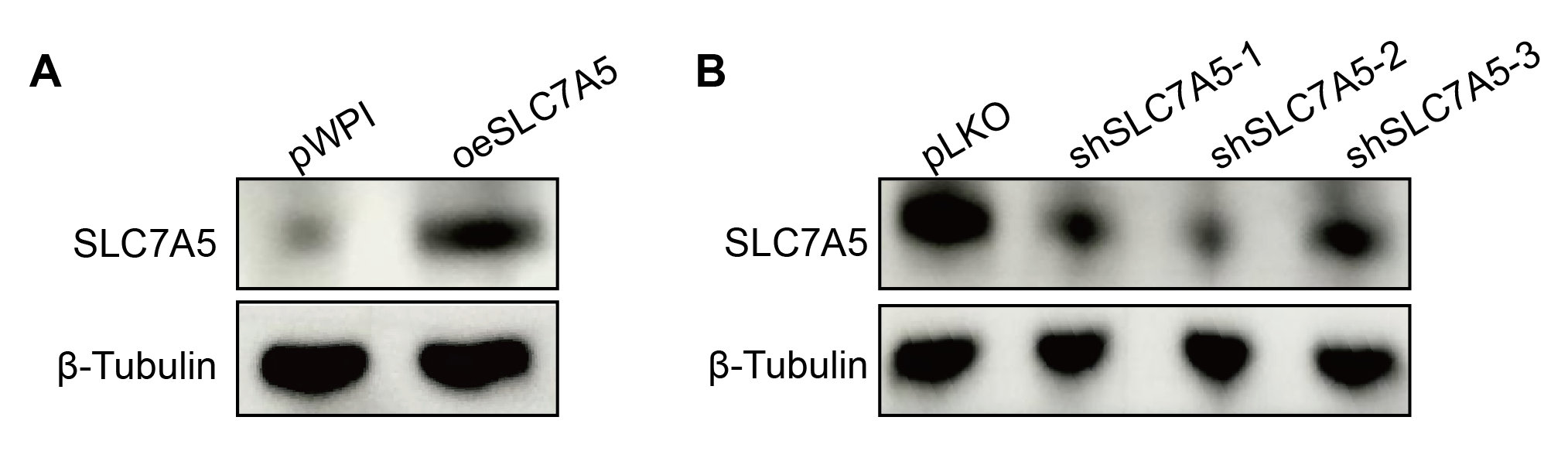

Supplement: Supplementary file 2 — Supplementary Material 2: Supplement figure 2. Western blot was validated the overexpression and knockdown efficiency of SLC7A5 [file 12935_2024_3365_MOESM2_ESM.tif]

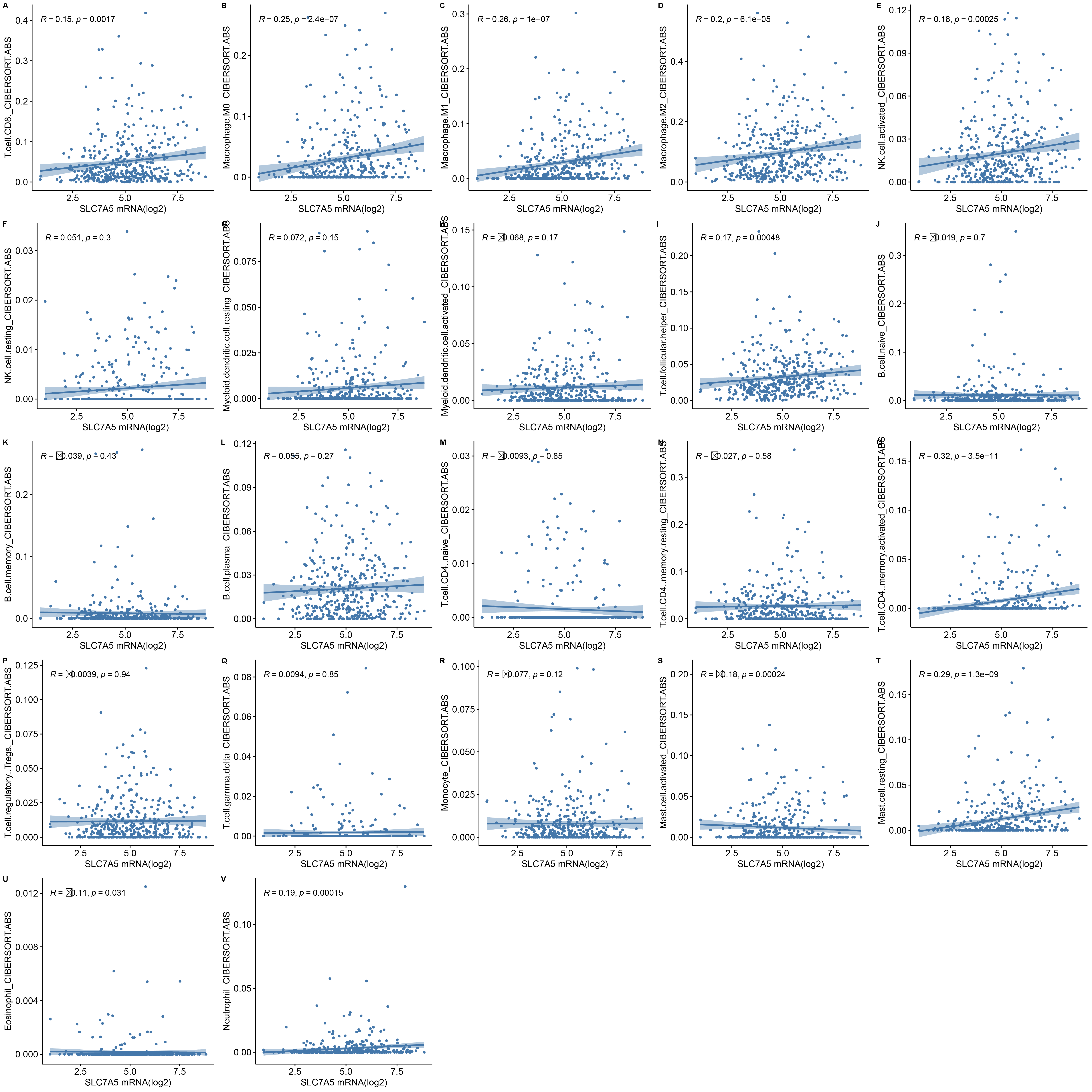

Supplement: Supplementary file 3 — Supplementary Material 3: Supplement figure 3. Based on the CIBERSORT-ABSA algorithm, SLC7A5 and tumor associated immune cells were correlated. Using spearman correlation analysis, the p value was calculated [file 12935_2024_3365_MOESM3_ESM.tif]

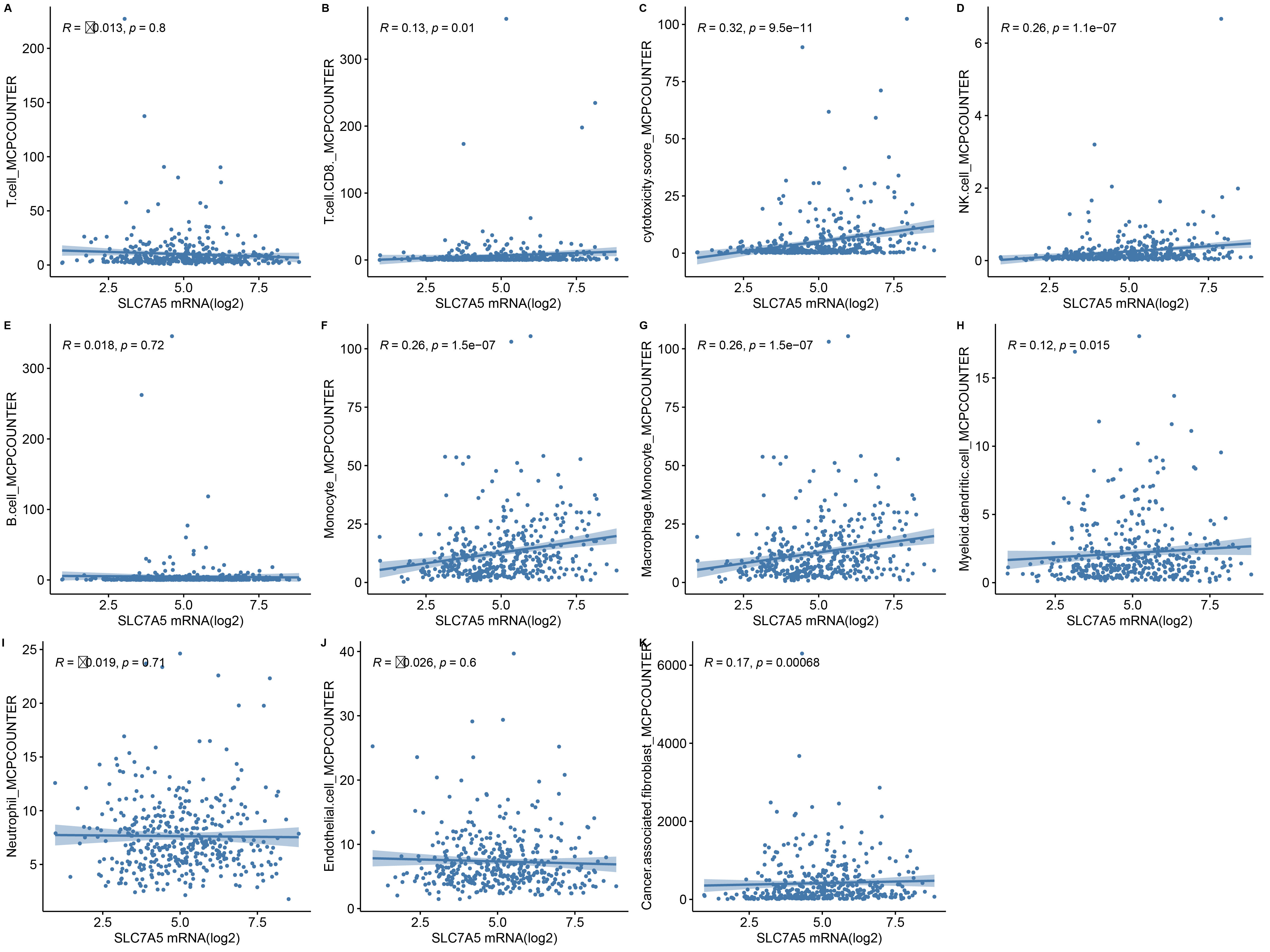

Supplement: Supplementary file 4 — Supplementary Material 4: Supplement figure 4. Based on the MCPCOUNTER algorithm, SLC7A5 and tumor associated immune cells were correlated. Using spearman correlation analysis, the p value was calculated [file 12935_2024_3365_MOESM4_ESM.tif]

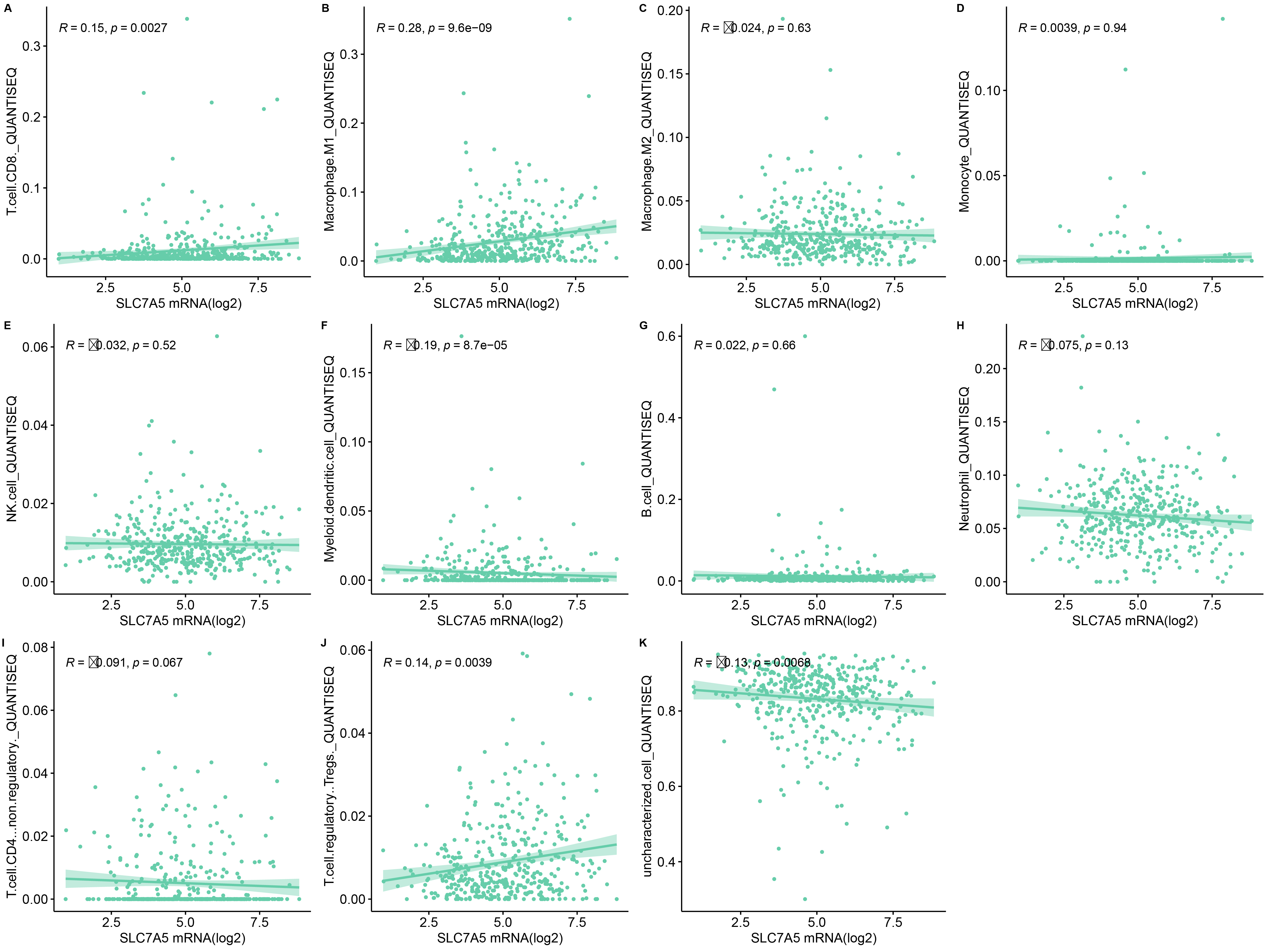

Supplement: Supplementary file 5 — Supplementary Material 5: Supplement figure 5. Based on the QUANTISEQ algorithm, SLC7A5 and tumor associated immune cells were correlated. Using spearman correlation analysis, the p value was calculated. [file 12935_2024_3365_MOESM5_ESM.tif]

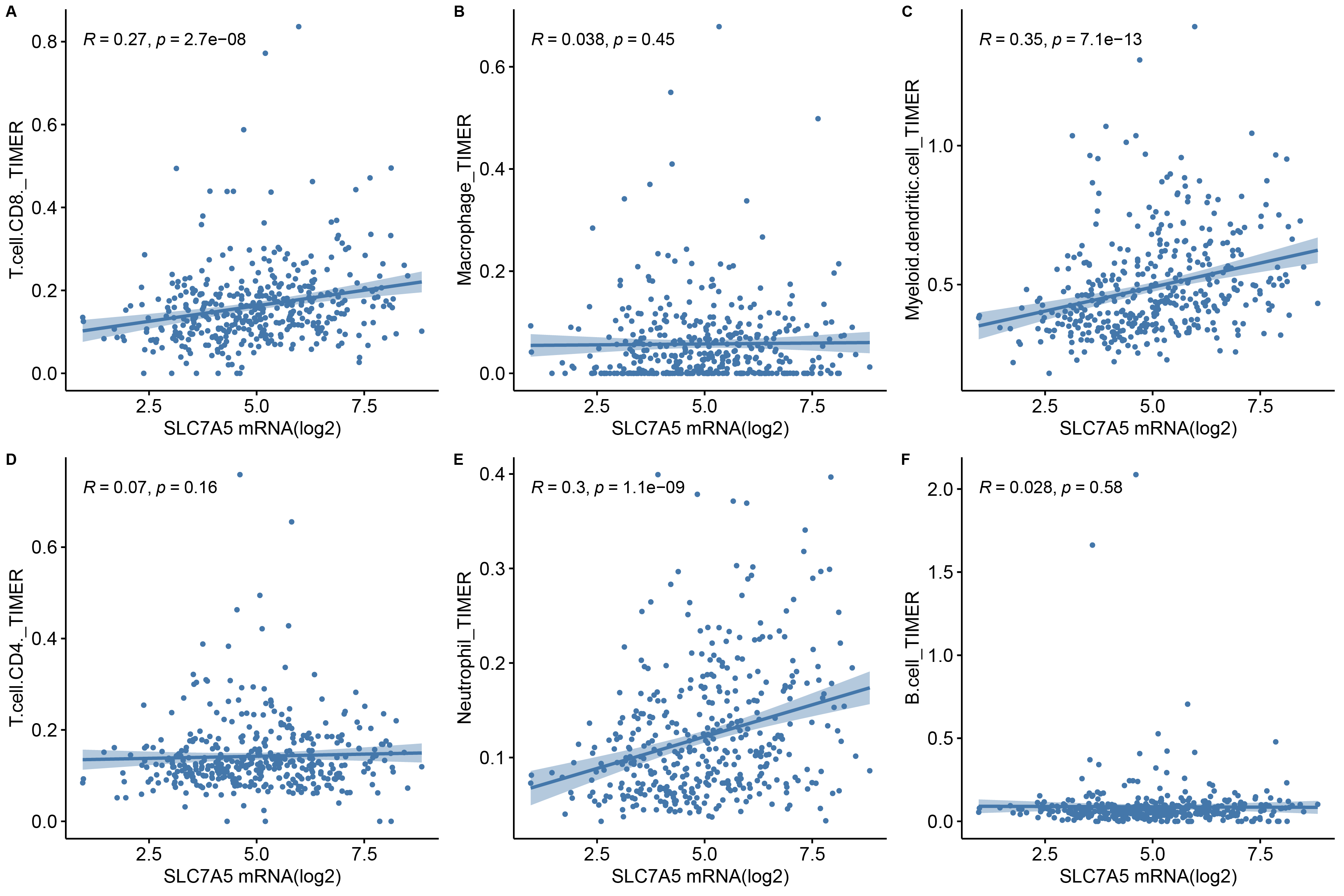

Supplement: Supplementary file 6 — Supplementary Material 6: Supplement figure 6. Based on the TIMER algorithm, SLC7A5 and tumor associated immune cells were correlated. Using spearman correlation analysis, the p value was calculated. [file 12935_2024_3365_MOESM6_ESM.tif]

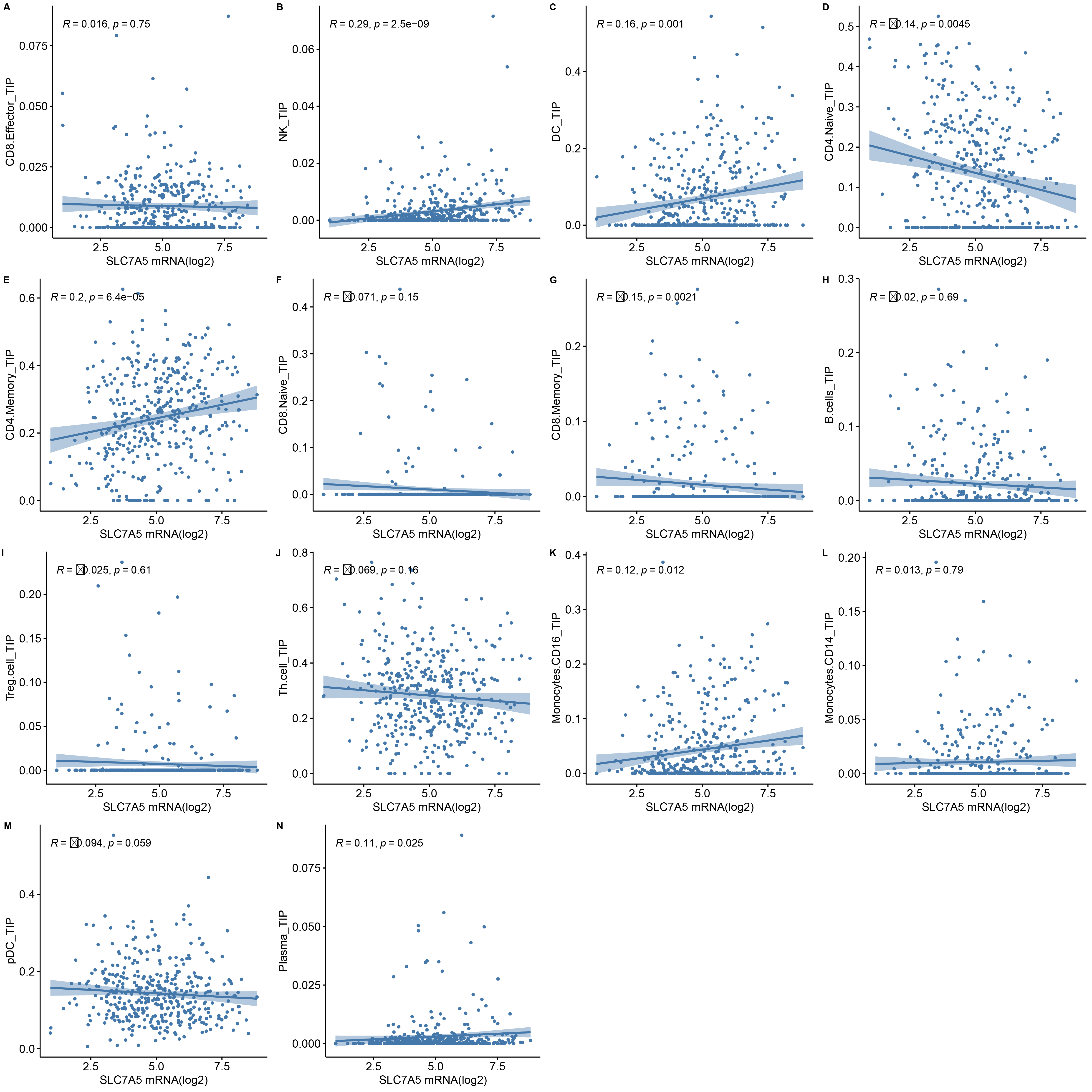

Supplement: Supplementary file 7 — Supplementary Material 7: Supplement figure 7. Based on the TIP algorithm, SLC7A5 and tumor associated immune cells were correlated. Using spearman correlation analysis, the p value was calculated. [file 12935_2024_3365_MOESM7_ESM.tif]

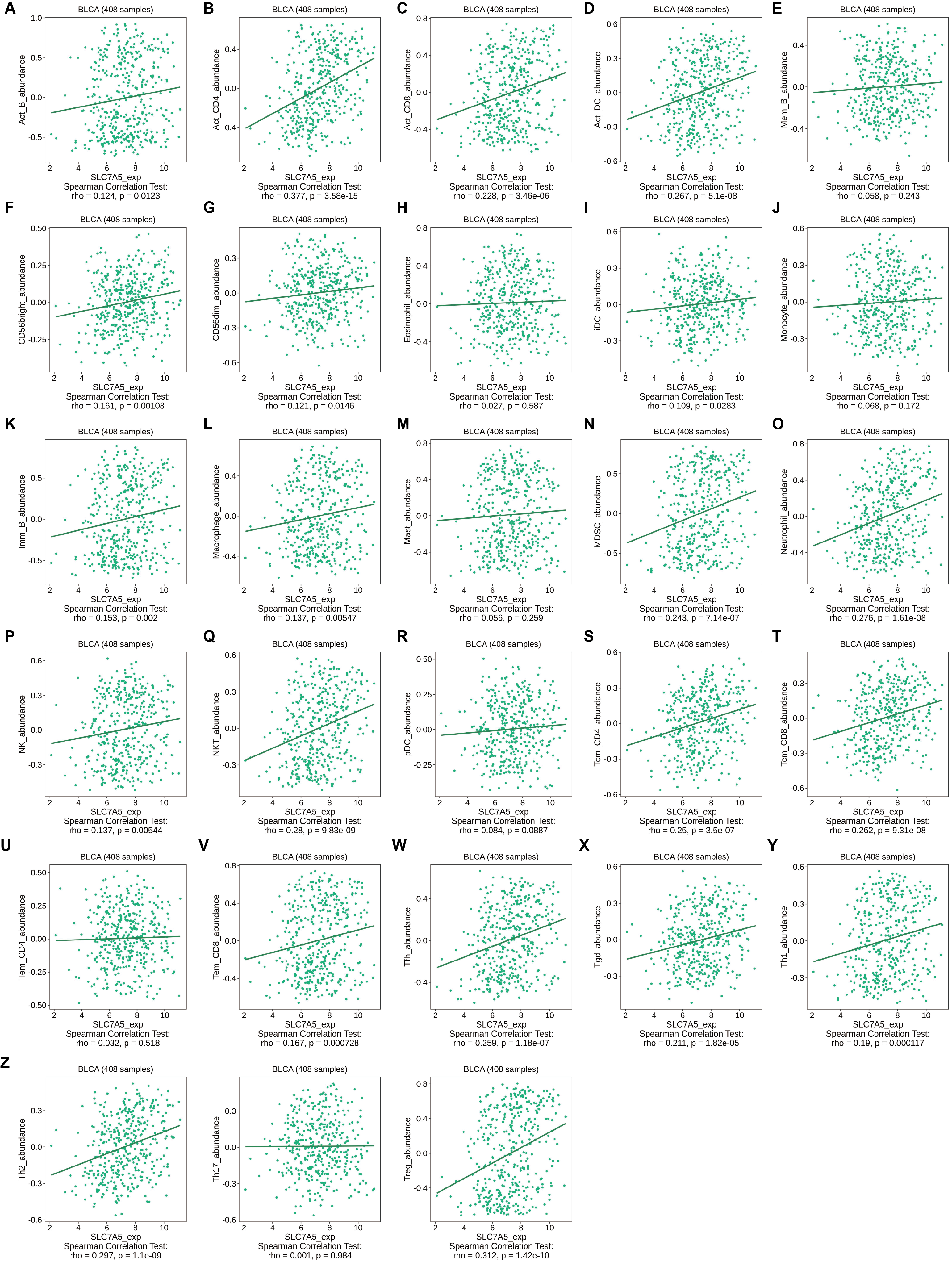

Supplement: Supplementary file 8 — Supplementary Material 8: Supplement figure 8. Based on the TISIDB algorithm, SLC7A5 and tumor associated immune cells were correlated. Using spearman correlation analysis, the p value was calculated [file 12935_2024_3365_MOESM8_ESM.tif]

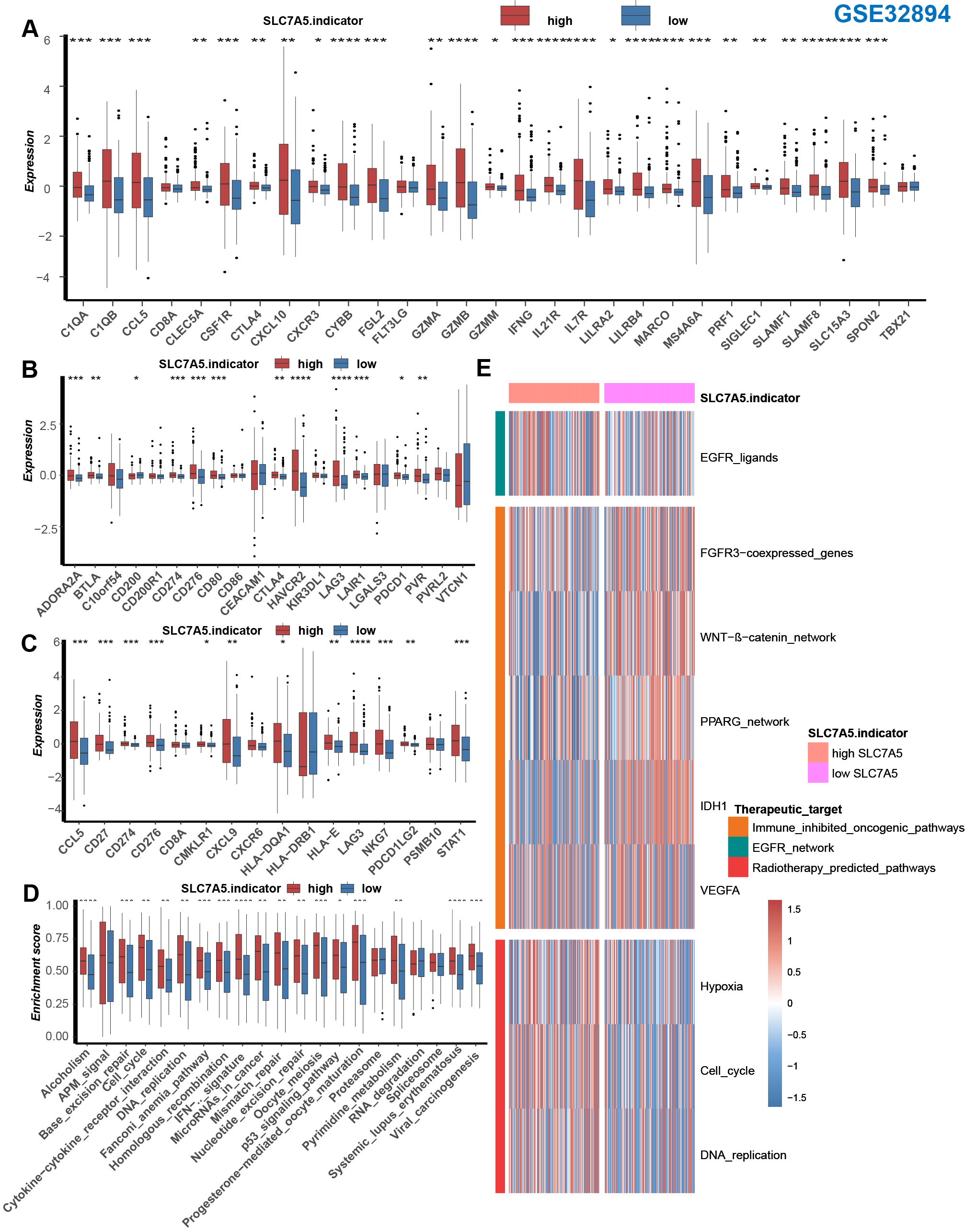

Supplement: Supplementary file 9 — Supplementary Material 9: Supplement figure 9. Based on the XCELL algorithm, SLC7A5 and tumor associated immune cells were correlated. Using spearman correlation analysis, the p value was calculated [file 12935_2024_3365_MOESM9_ESM.tif]

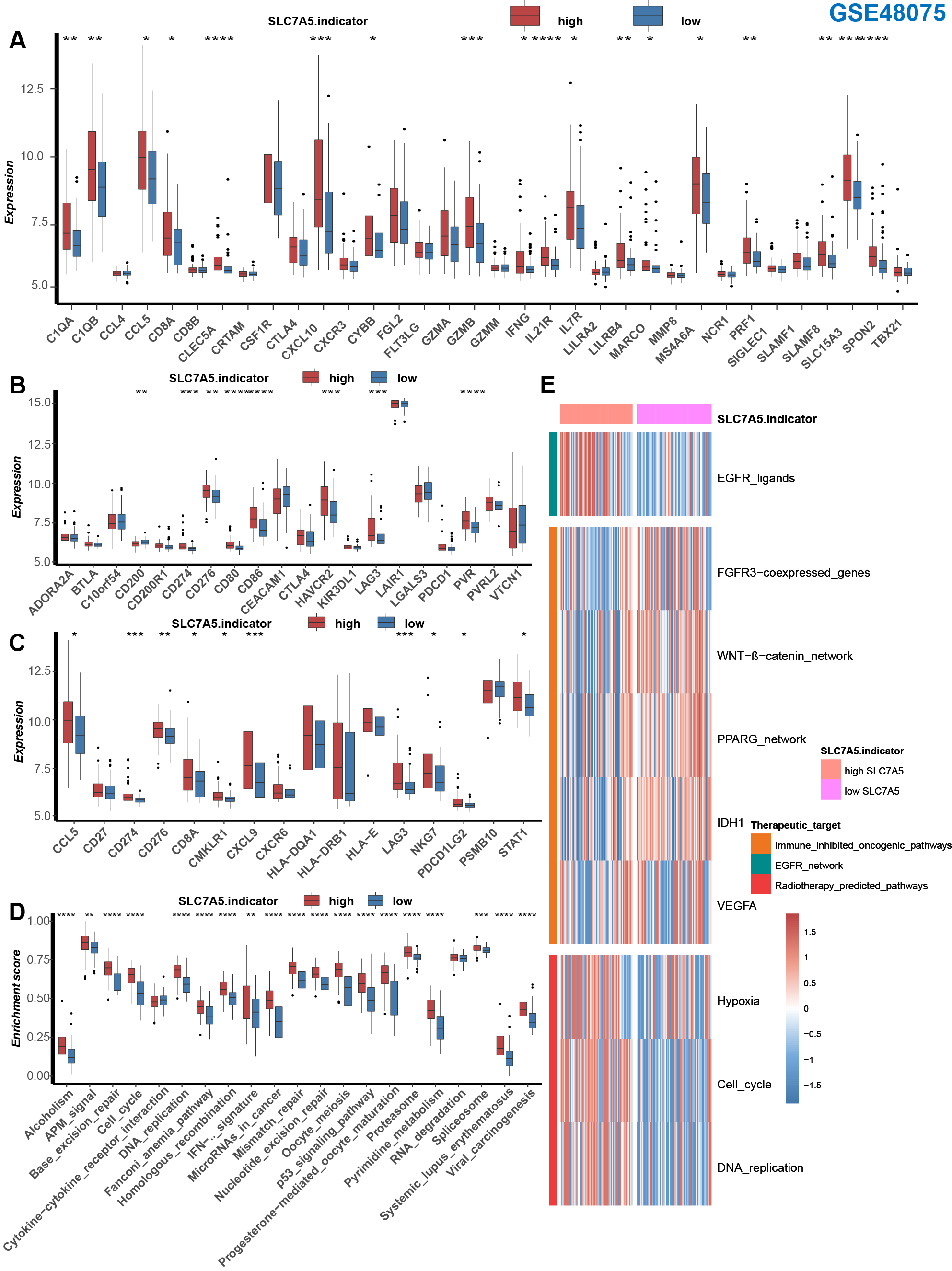

Supplement: Supplementary file 11 — Supplementary Material 11: Supplement figure 11. In GSE48075, SLC7A5 plays a role in predicting immune phenotypes and therapeutic responses. A-D. Correlation between SLC7A5 and inhibitory immune checkpoints, enrichment scores of immunotherapy and effector genes of tumor associated immune cells predicted signatures and tumor inflammation signature in BLCA. E. The correlation between SLC7A5 and the enrichment scores of several therapeutic signatures, including targeted therapy and radiation therapy. Asterisks indicate statistically significant p values calculated by the Mann-Whitney U test. *P＜0.05; **P＜0.01; ***P＜0.001 [file 12935_2024_3365_MOESM11_ESM.tif]

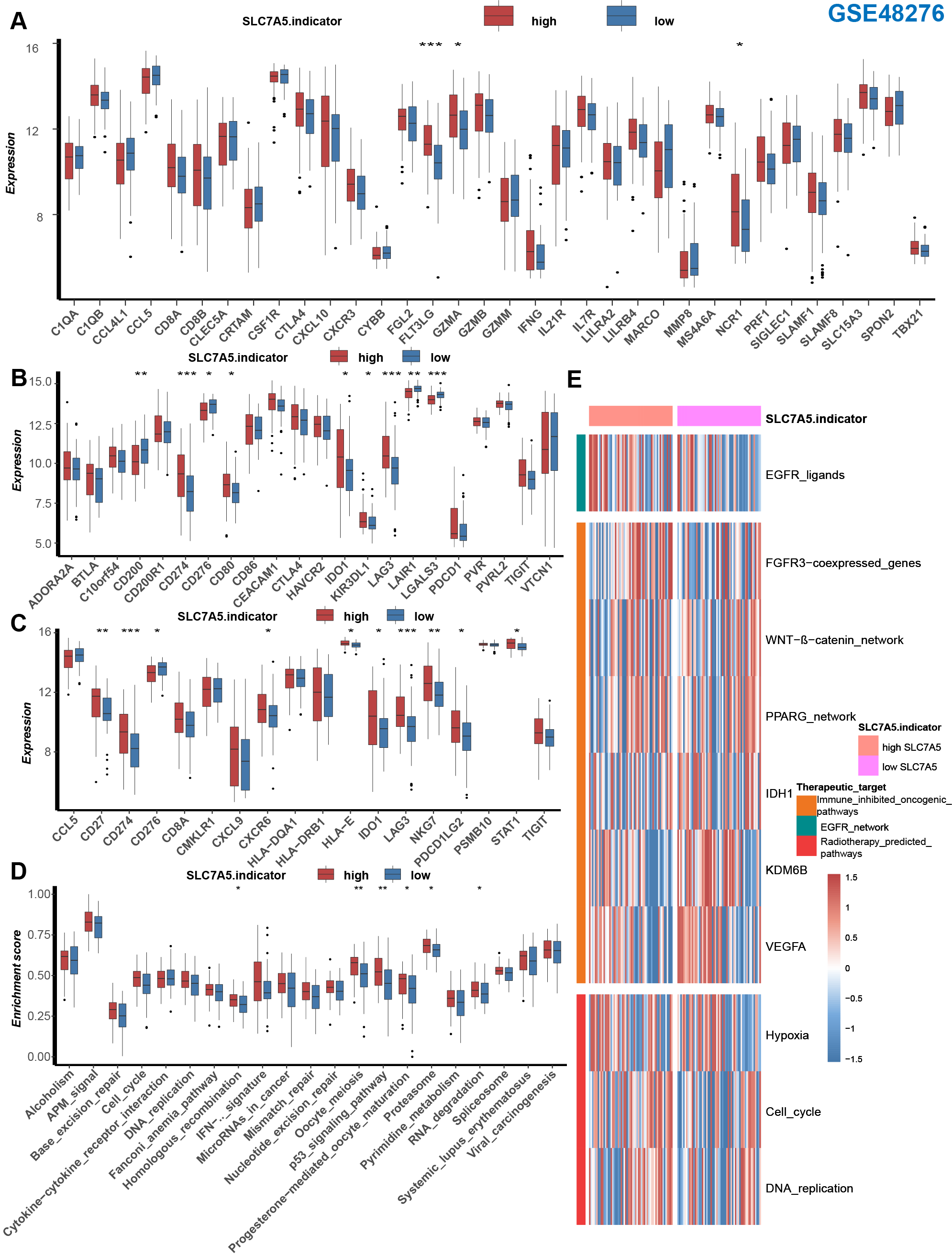

Supplement: Supplementary file 12 — Supplementary Material 12: Supplement figure 12. In GSE48276, SLC7A5 plays a role in predicting immune phenotypes and therapeutic responses. A-D. Correlation between SLC7A5 and inhibitory immune checkpoints, enrichment scores of immunotherapy and effector genes of tumor associated immune cells predicted signatures and tumor inflammation signature in BLCA. E. The correlation between SLC7A5 and the enrichment scores of several therapeutic signatures, including targeted therapy and radiation therapy. Asterisks indicate statistically significant p values calculated by the Mann-Whitney U test. *P＜0.05; **P＜0.01; ***P＜0.001 [file 12935_2024_3365_MOESM12_ESM.tif]

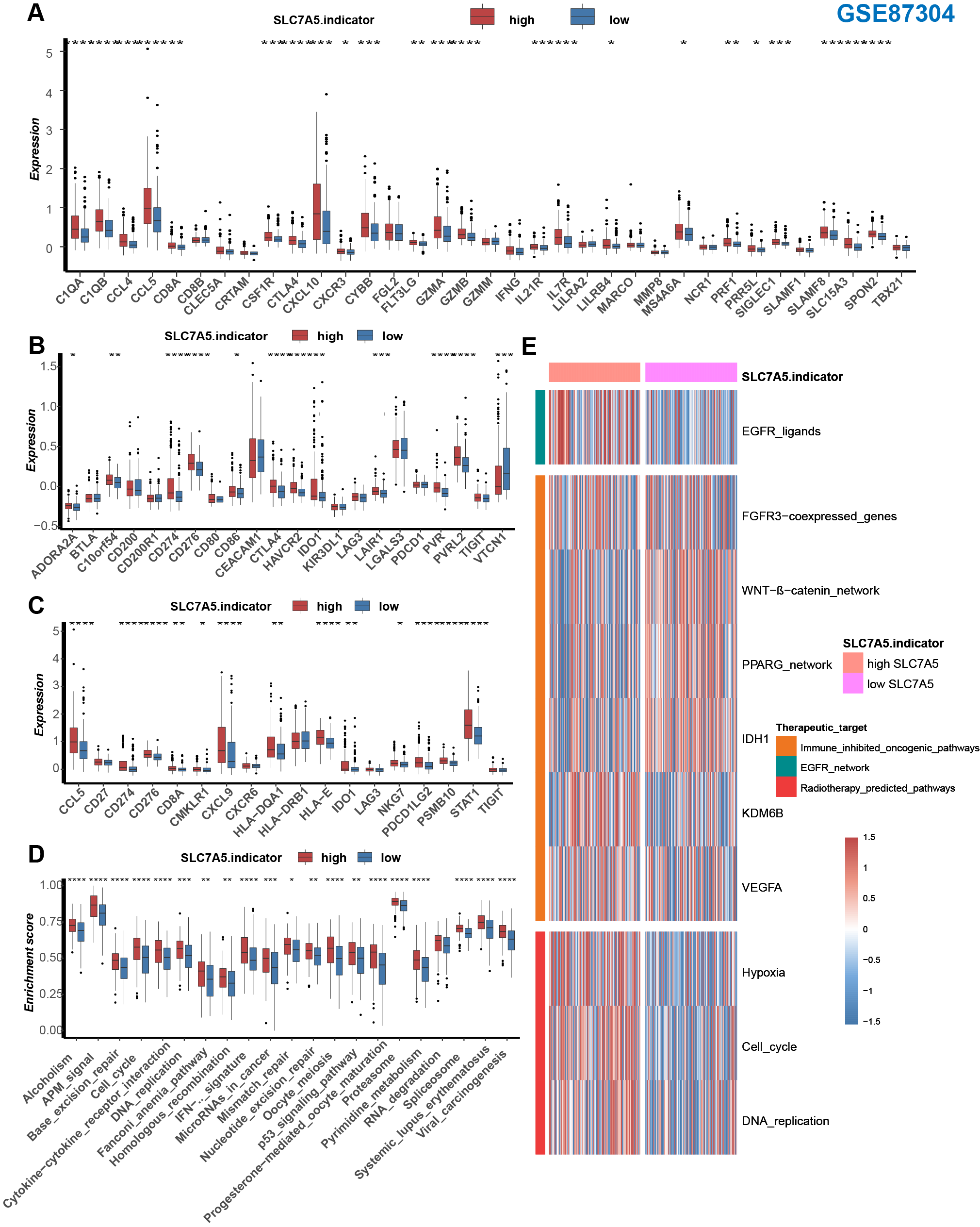

Supplement: Supplementary file 13 — Supplementary Material 13: Supplement figure 13. In GSE87304, SLC7A5 plays a role in predicting immune phenotypes and therapeutic responses. A-D. Correlation between SLC7A5 and inhibitory immune checkpoints, enrichment scores of immunotherapy and effector genes of tumor associated immune cells predicted signatures and tumor inflammation signature in BLCA. E. The correlation between SLC7A5 and the enrichment scores of several therapeutic signatures, including targeted therapy and radiation therapy. Asterisks indicate statistically significant p values calculated by the Mann-Whitney U test. *P＜0.05; **P＜0.01; ***P＜0.001 [file 12935_2024_3365_MOESM13_ESM.tif]

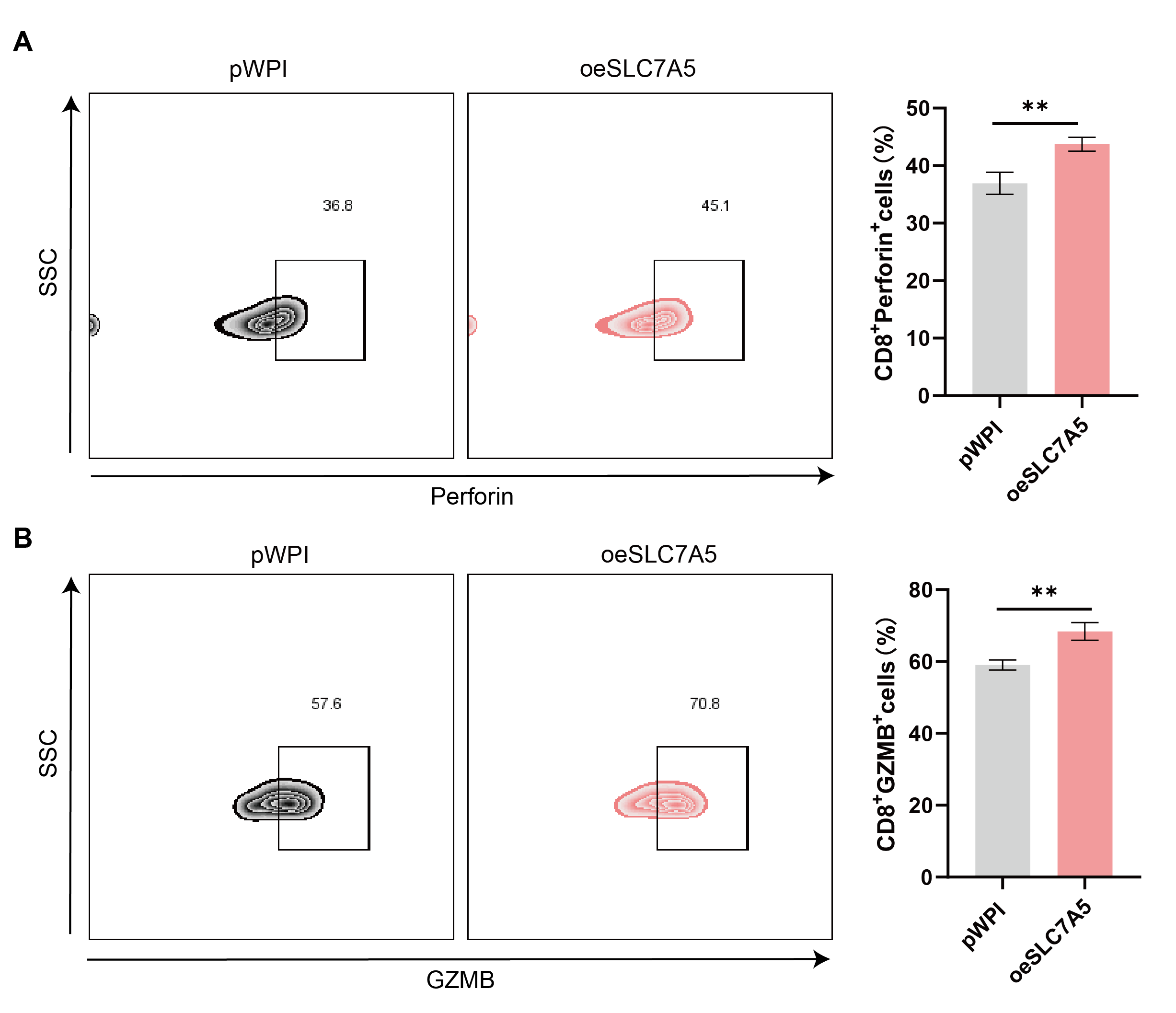

Supplement: Supplementary file 14 — Supplementary Material 14: Supplement figure 14. Results of coculture experiment between T cells and tumor cells. *P＜0.05; **P＜0.01; ***P＜0.001 [file 12935_2024_3365_MOESM14_ESM.tif]

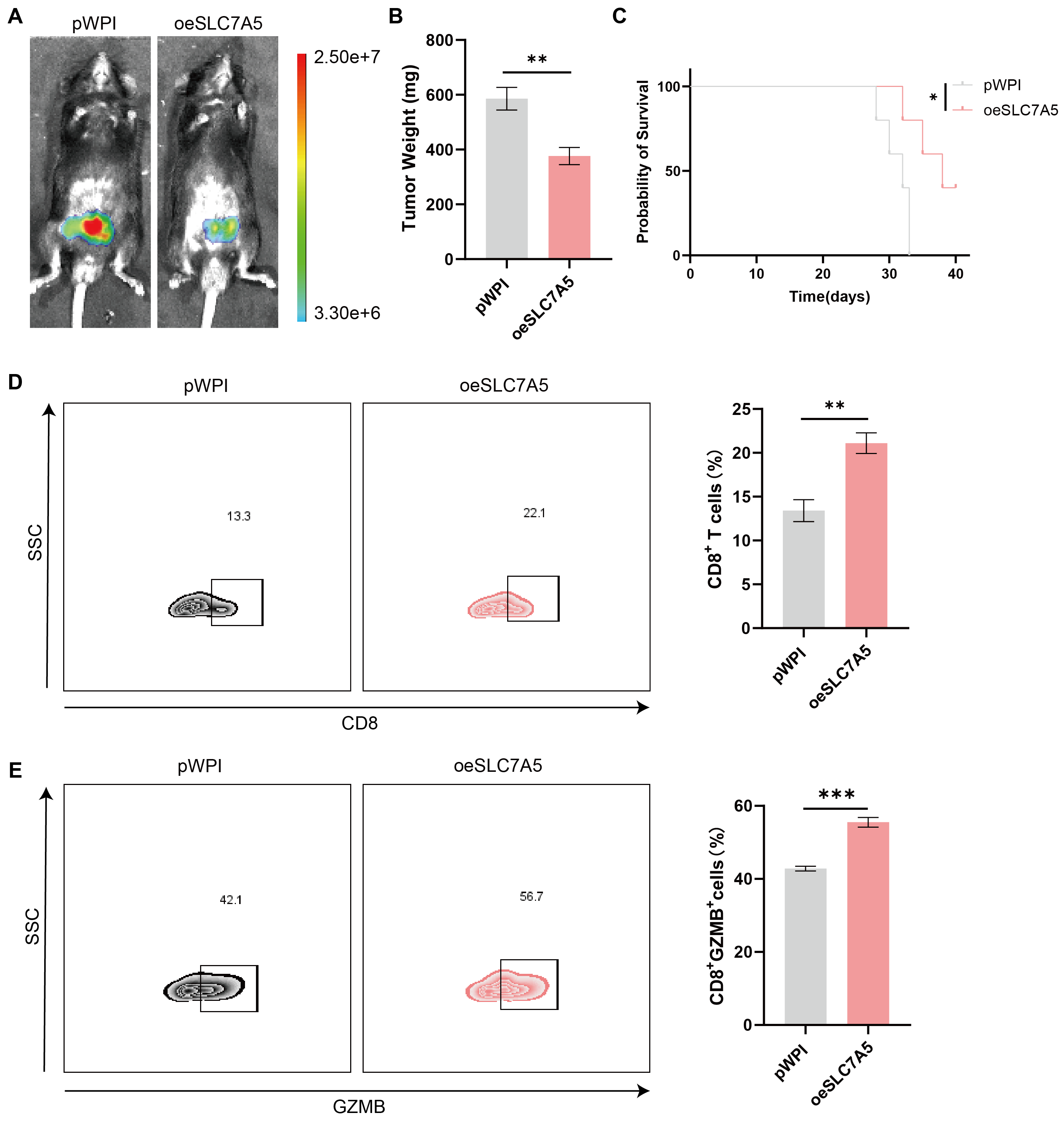

Supplement: Supplementary file 15 — Supplementary Material 15: Supplement figure 15. In vivo animal experiments validate the tumor immunological role of SLC7A5. *P＜0.05; **P＜0.01; ***P＜0.001 [file 12935_2024_3365_MOESM15_ESM.tif]

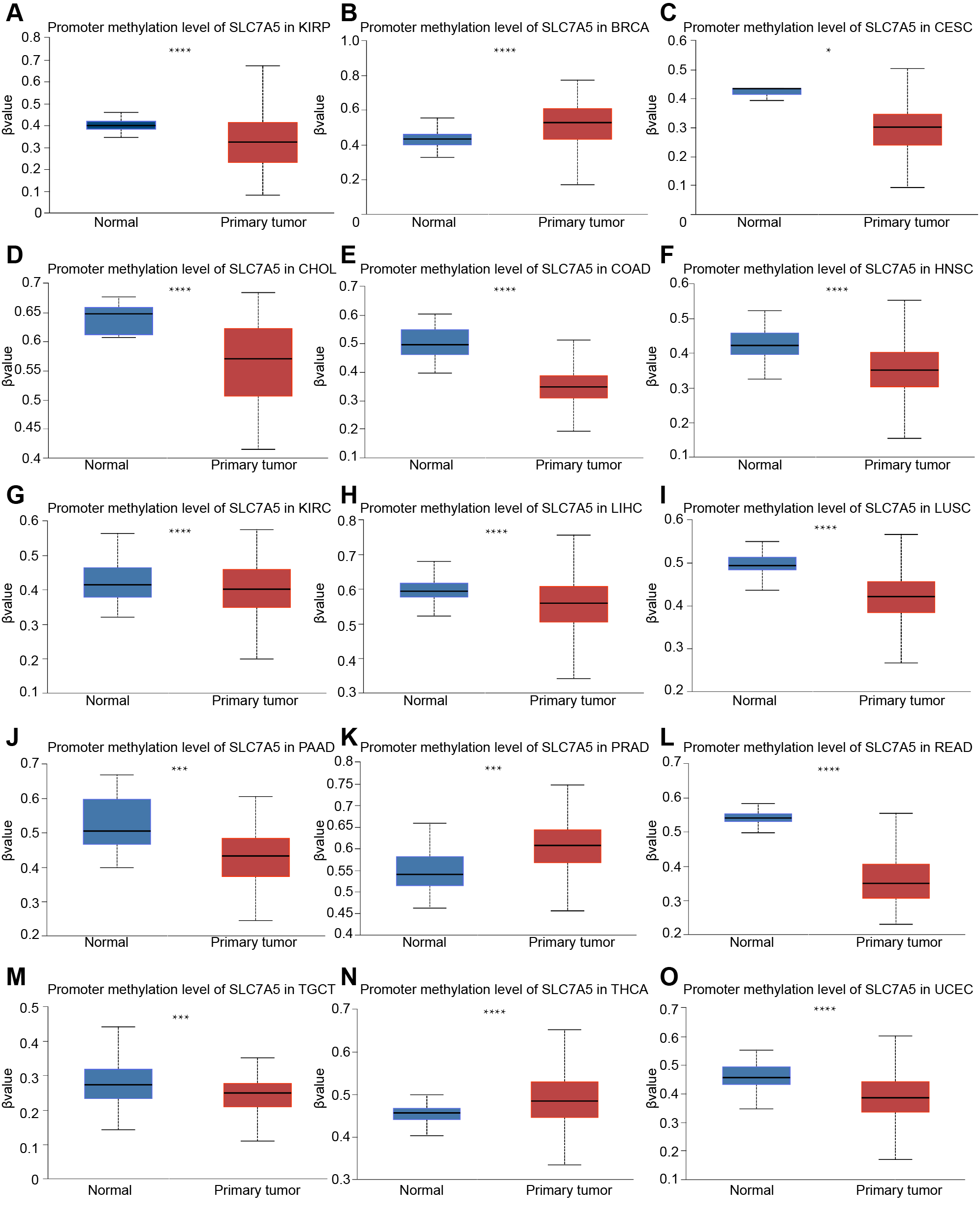

Supplement: Supplementary file 16 — Supplementary Material 16: Supplement figure 16. Promoter methylation level of SLC7A5 in pan-cancers. *P＜0.05; **P＜0.01; ***P＜0.001 [file 12935_2024_3365_MOESM16_ESM.tif]

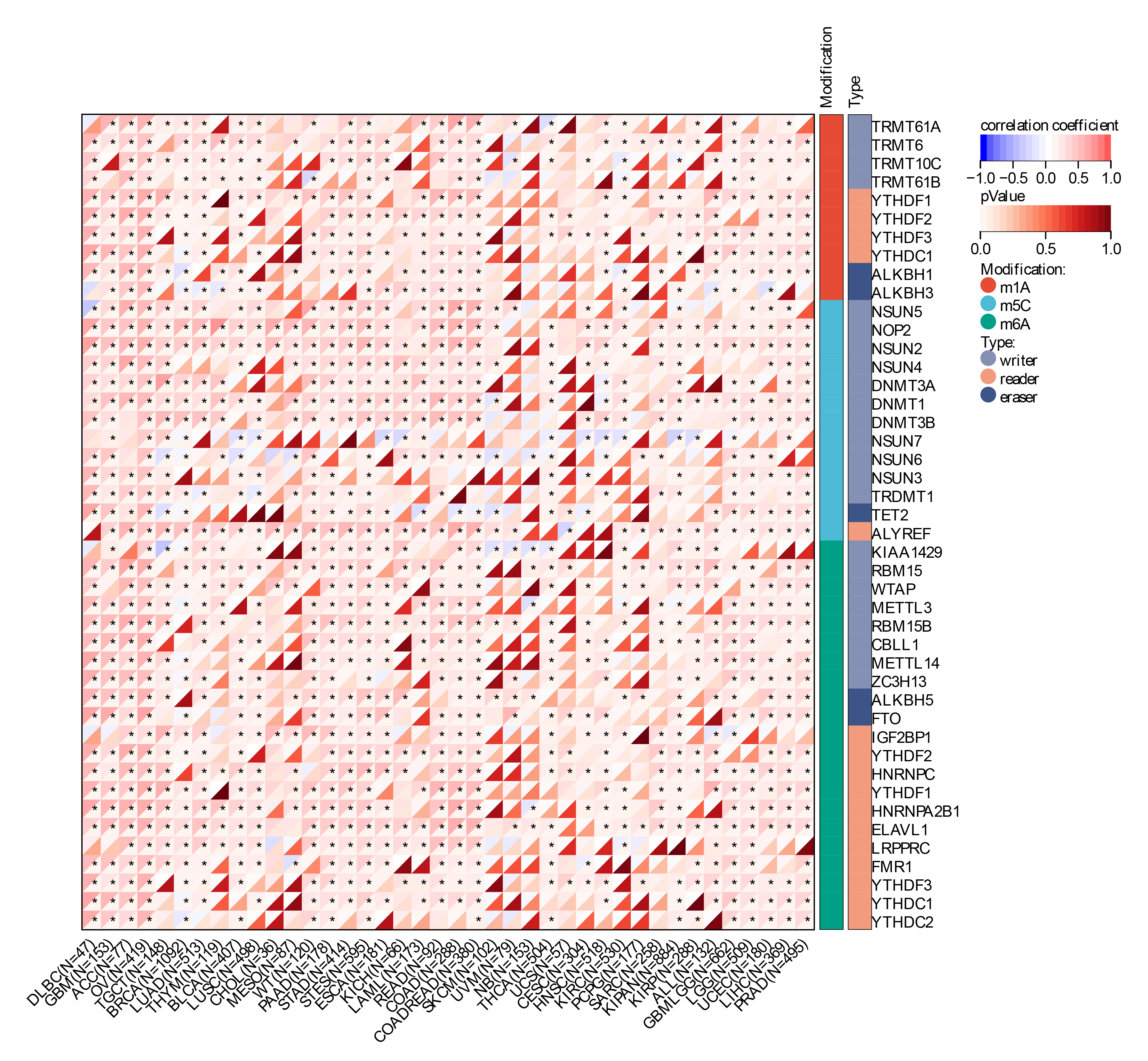

Supplement: Supplementary file 17 — Supplementary Material 17: Supplement figure 17. Correlation between SLC7A5 and m1A, m5C and m6A related genes [file 12935_2024_3365_MOESM17_ESM.tif]

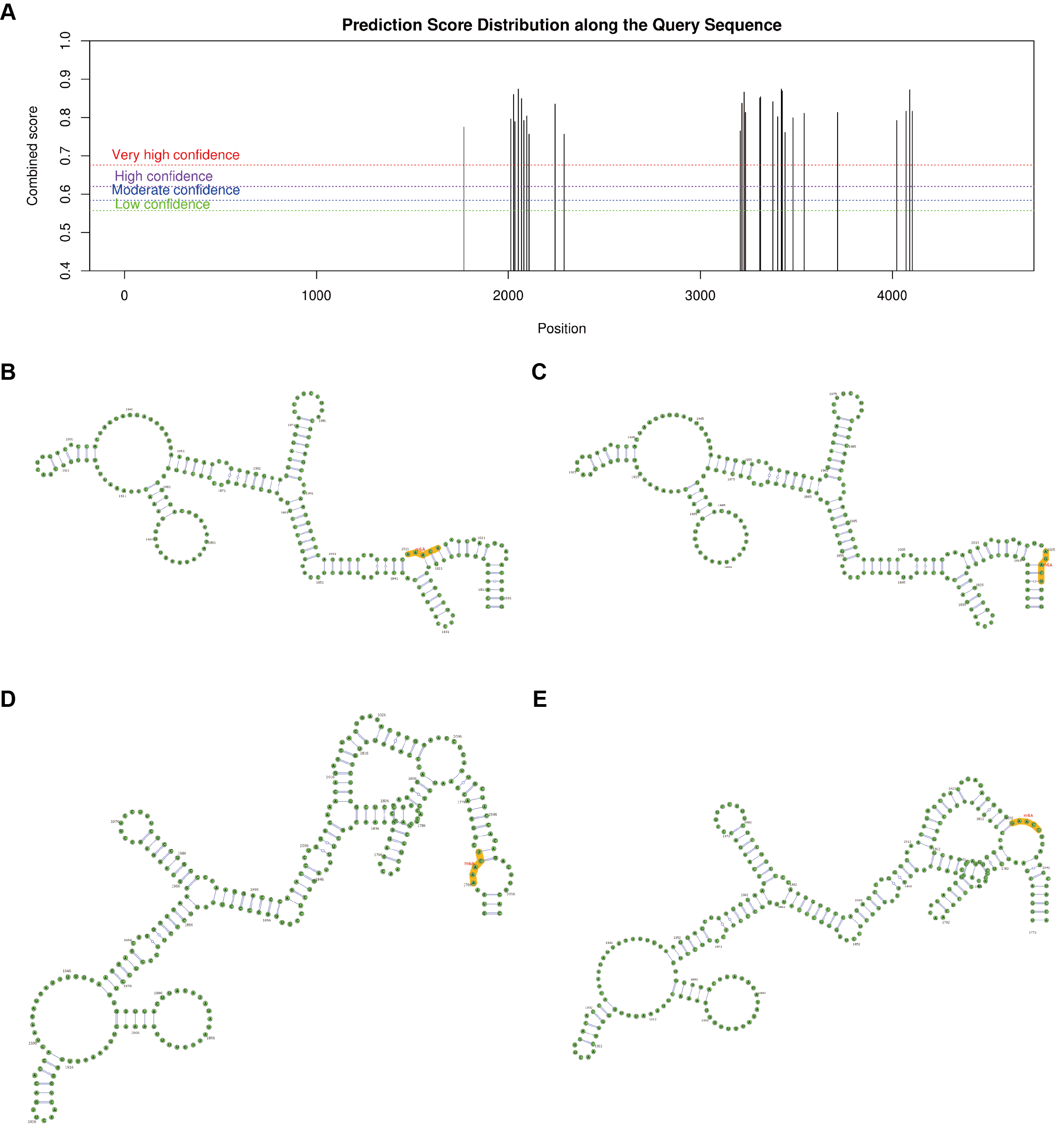

Supplement: Supplementary file 18 — Supplementary Material 18: Supplement figure 18. SRAMP was used to predicts the m6a sites of SLC7A5 [file 12935_2024_3365_MOESM18_ESM.tif]

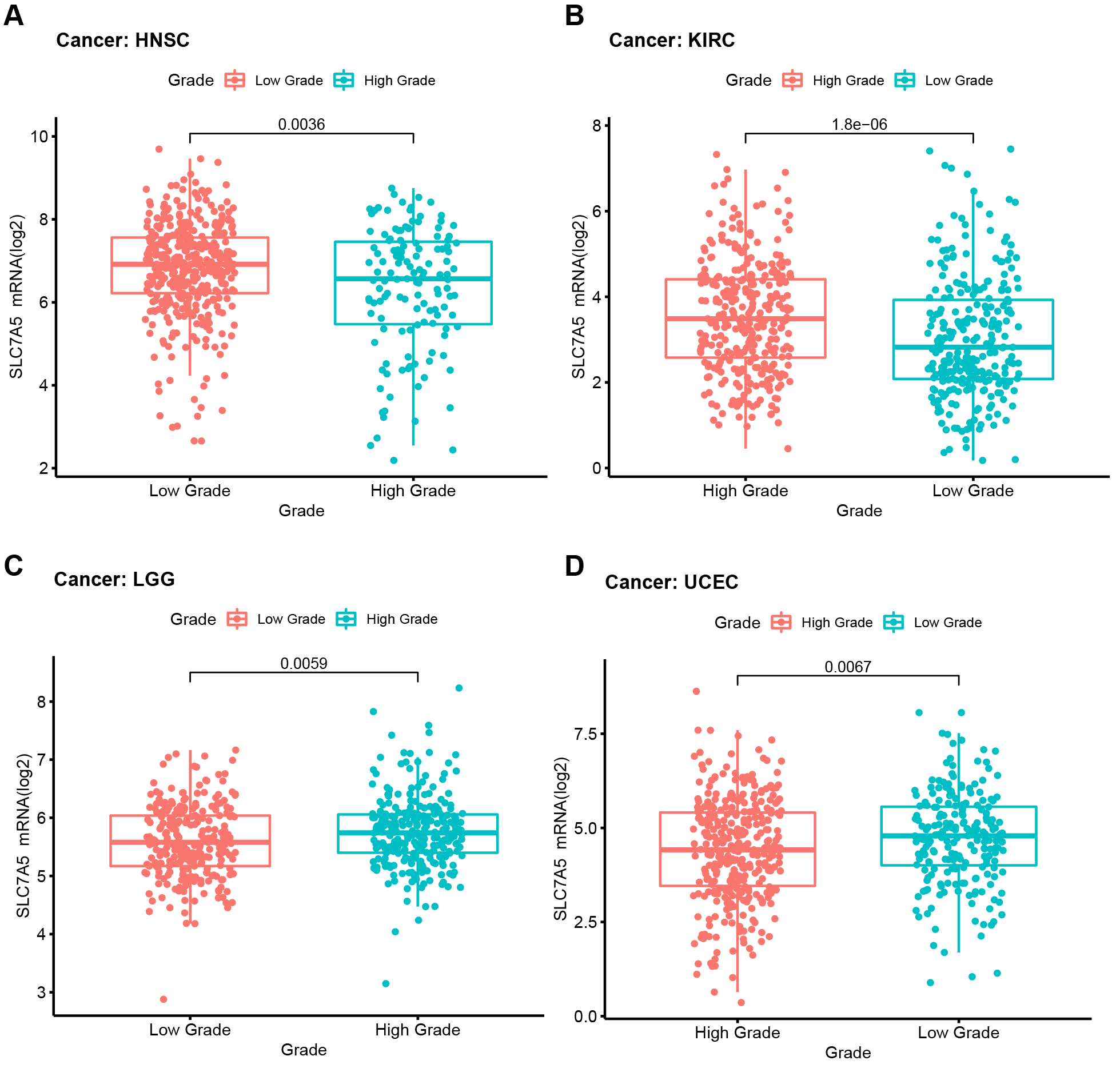

Supplement: Supplementary file 19 — Supplementary Material 19: Supplement figure 19. In pan-cancers, TCGA data was used to analyze the expression of the SLC7A5 gene based on grade (high grade, low grade). [file 12935_2024_3365_MOESM19_ESM.tif]

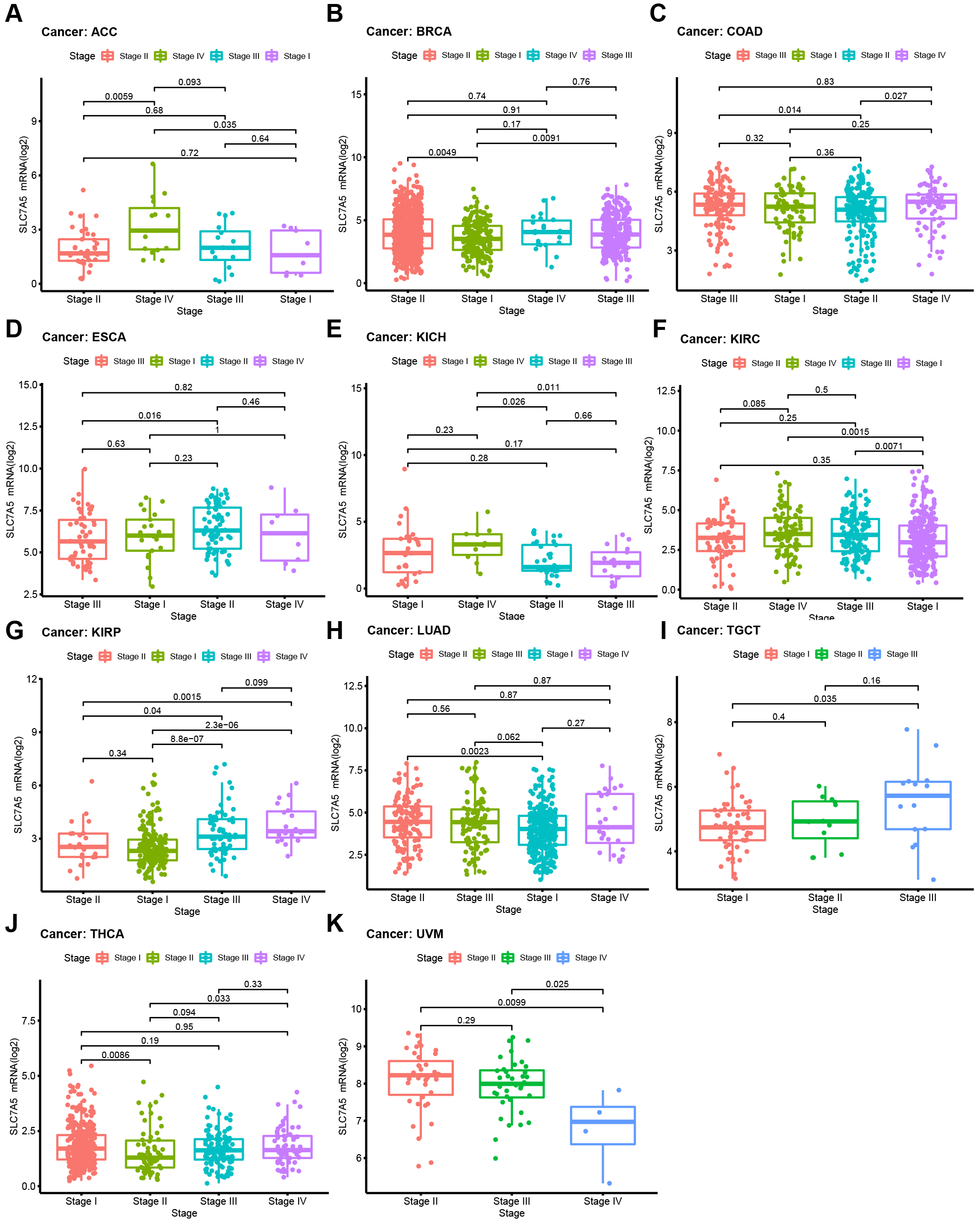

Supplement: Supplementary file 20 — Supplementary Material 20: Supplement figure 20. In pan-cancers, TCGA data was used to analyze the expression of the SLC7A5 gene based on stage (stage I, stage II, stage III, and stage IV) [file 12935_2024_3365_MOESM20_ESM.tif]

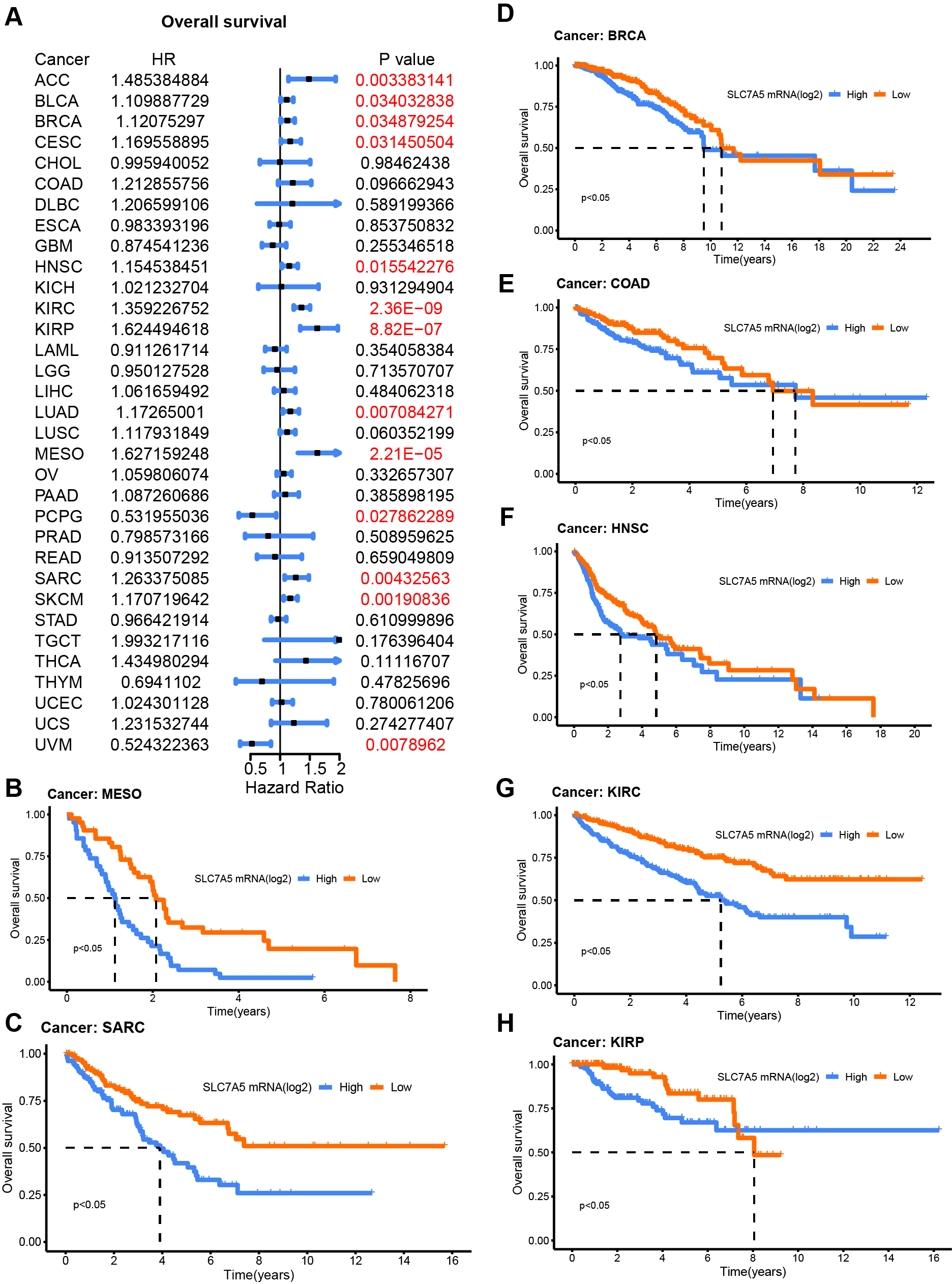

Supplement: Supplementary file 21 — Supplementary Material 21: Supplement figure 21. Prognostic analysis of SLC7A5 for overall survival in pan-cancers. A. Using a univariate Cox regression model, the prognostic significance of SLC7A5 in pan-cancers was assessed. Hazard ratio >1 represented a risk factor, and hazard ratio [file 12935_2024_3365_MOESM21_ESM.tif]

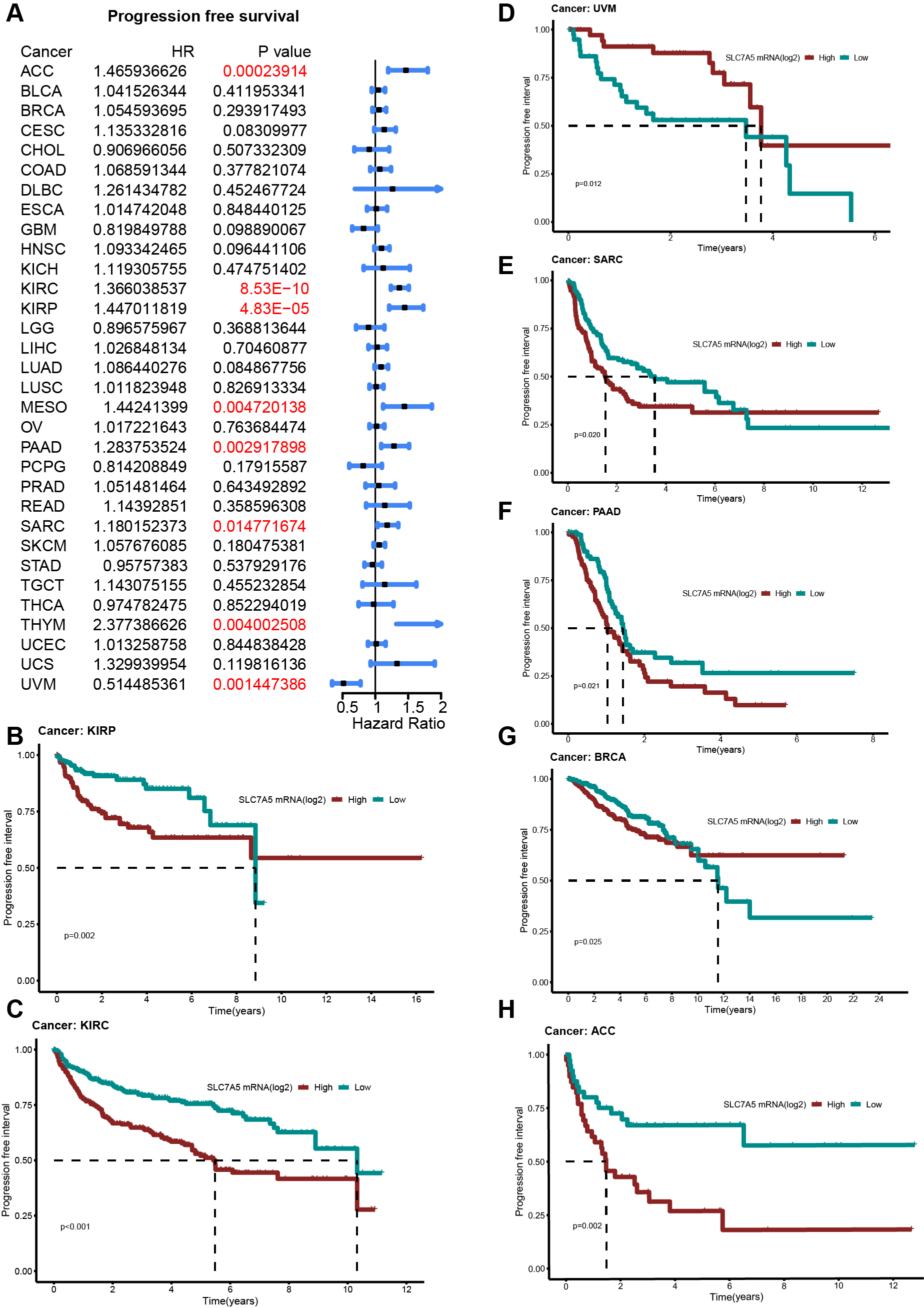

Supplement: Supplementary file 22 — Supplementary Material 22: Supplement figure 22. Prognostic analysis of SLC7A5 for progression free survival in pan-cancers. A. Using a univariate Cox regression model, the prognostic significance of SLC7A5 in pan-cancers was assessed. Hazard ratio >1 represented a risk factor, and hazard ratio [file 12935_2024_3365_MOESM22_ESM.tif]

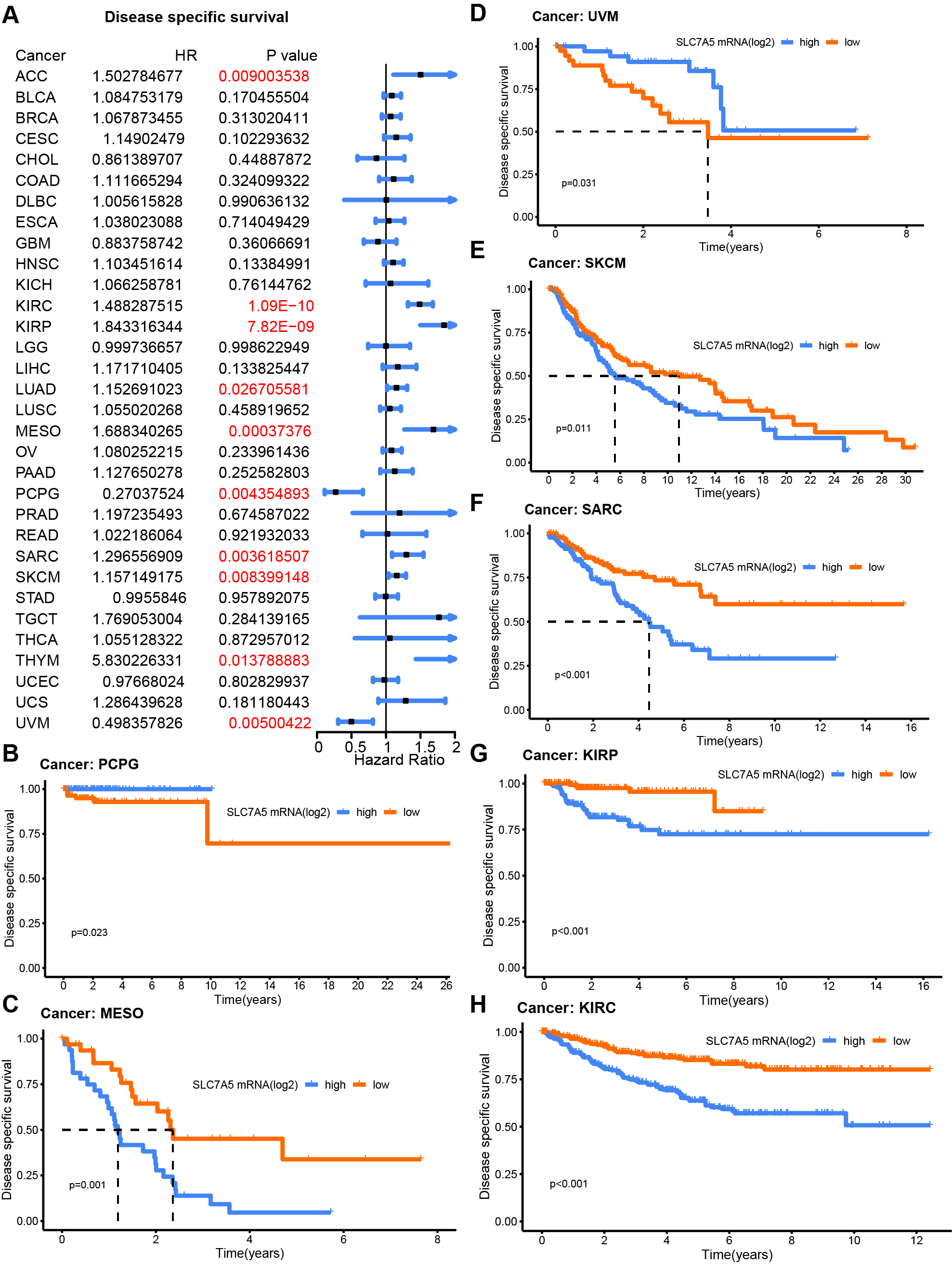

Supplement: Supplementary file 23 — Supplementary Material 23: Supplement figure 23. Prognostic analysis of SLC7A5 for disease specific survival in pan-cancers. A. Using a univariate Cox regression model, the prognostic significance of SLC7A5 in pan-cancers was assessed. Hazard ratio >1 represented a risk factor, and hazard ratio [file 12935_2024_3365_MOESM23_ESM.tif]

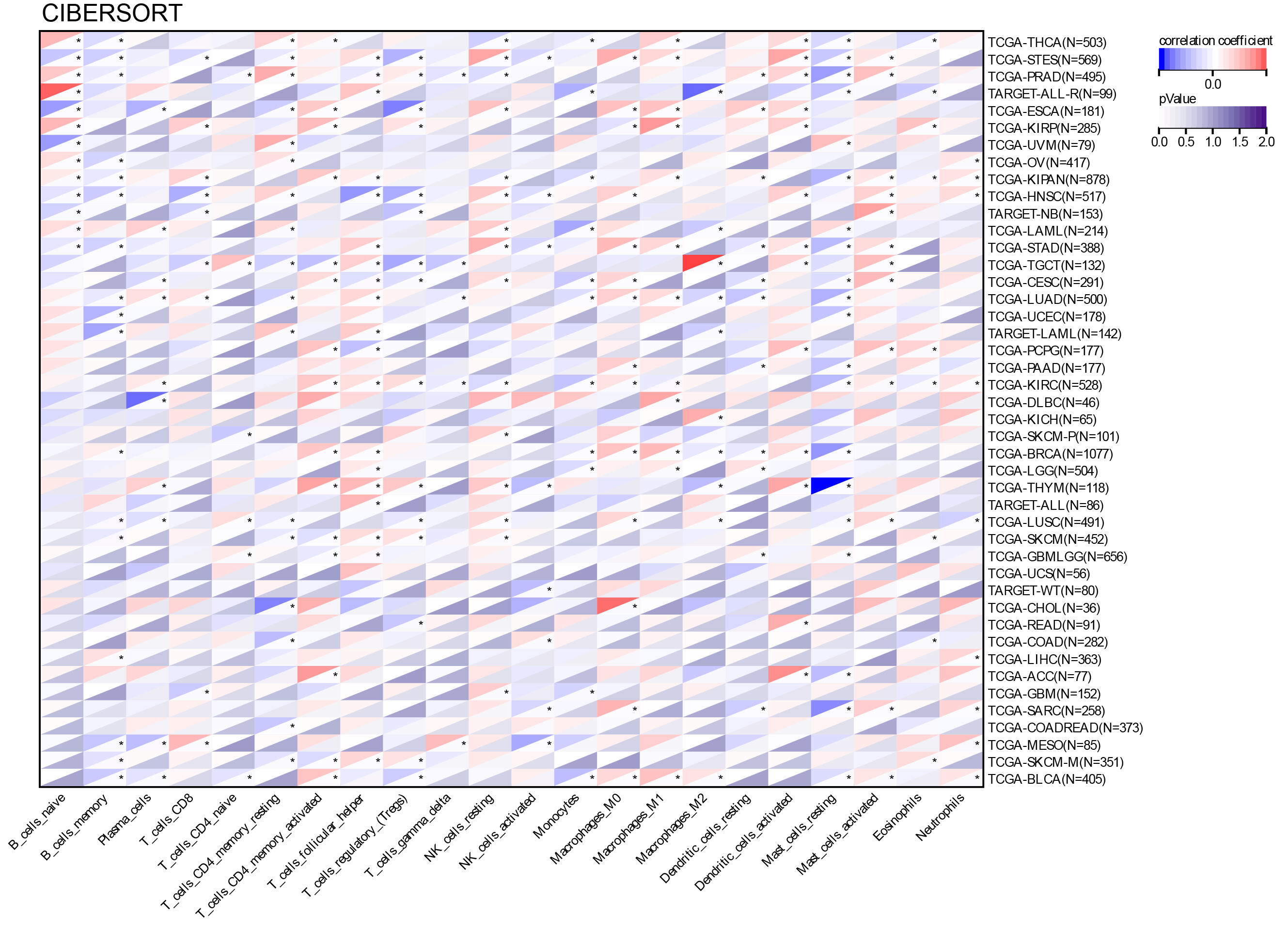

Supplement: Supplementary file 24 — Supplementary Material 24: Supplement figure 24. The CIBERSORT algorithm was used to calculate a correlation between SLC7A5 and tumor-associated immune cells. Correlation coefficient is indicated by the color. When an asterisk appears, it indicates a statistically significant p-value based on spearman correlation analysis. *P＜0.05; **P＜0.01; ***P＜0.001 [file 12935_2024_3365_MOESM24_ESM.tif]

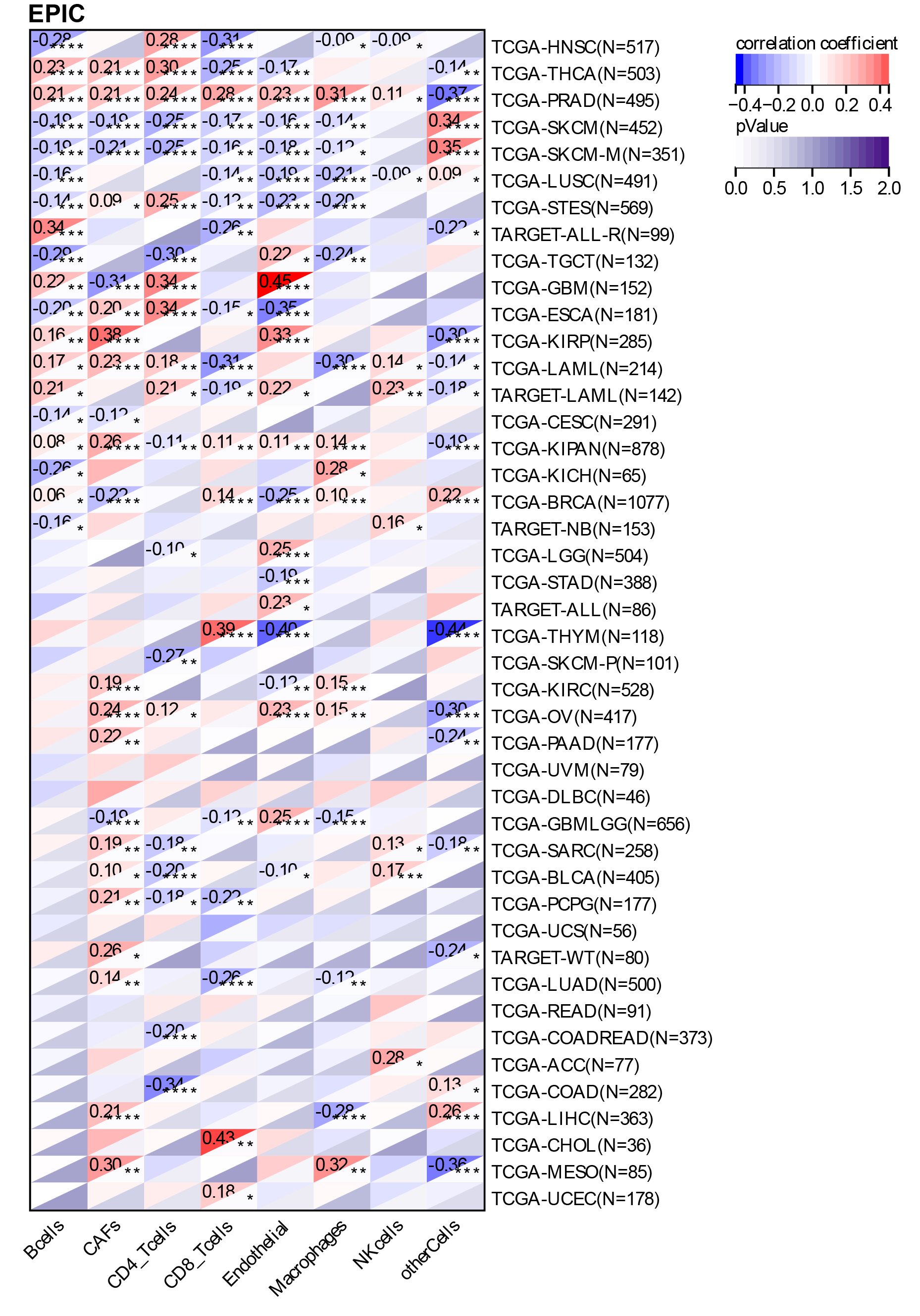

Supplement: Supplementary file 25 — Supplementary Material 25: Supplement figure 25. The EPIC algorithm was used to calculate a correlation between SLC7A5 and tumor-associated immune cells. Correlation coefficient is indicated by the color. When an asterisk appears, it indicates a statistically significant p-value based on spearman correlation analysis. *P＜0.05; **P＜0.01; ***P＜0.001 [file 12935_2024_3365_MOESM25_ESM.tif]

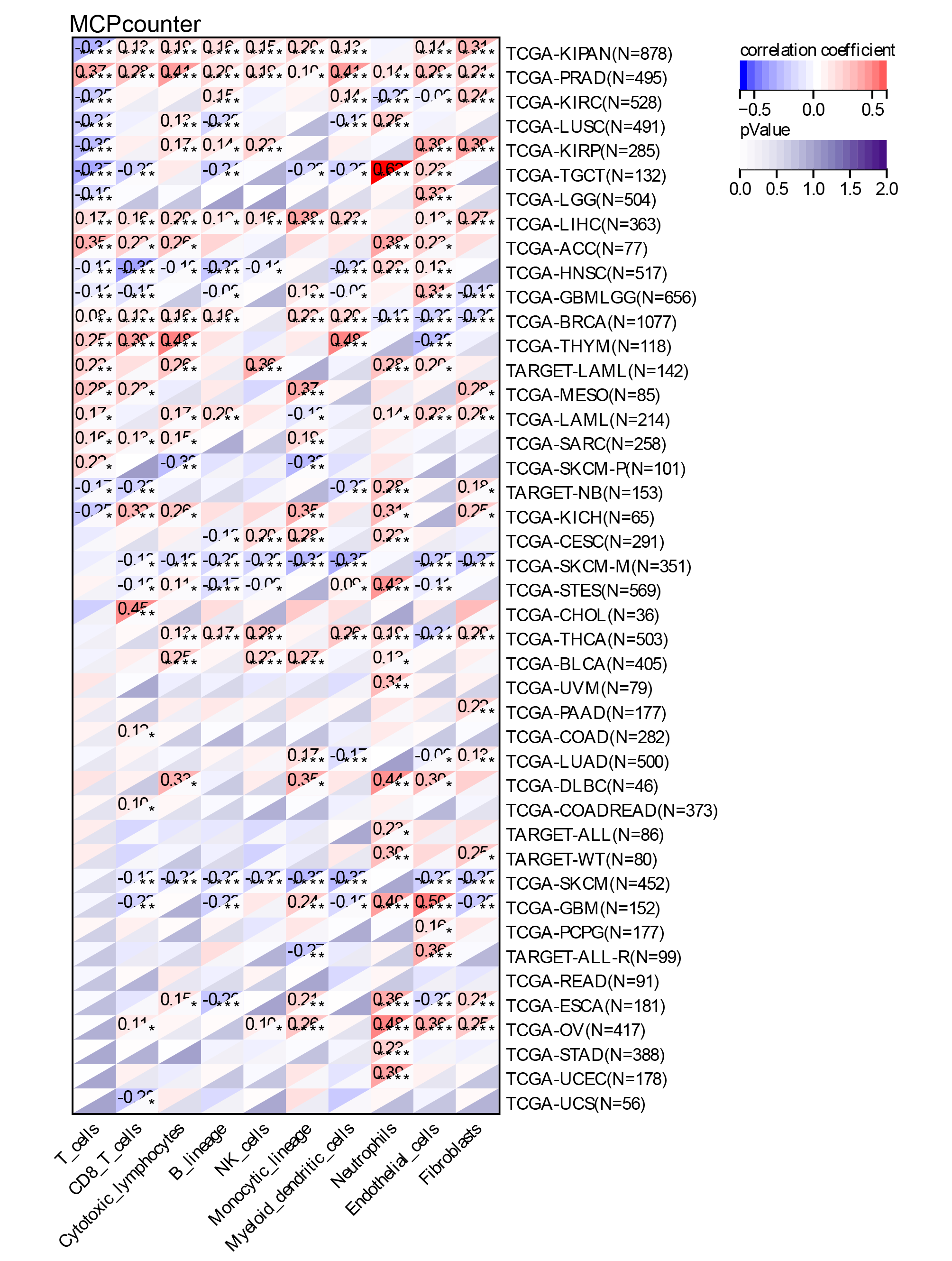

Supplement: Supplementary file 26 — Supplementary Material 26: Supplement figure 26. The MCPcounter algorithm was used to calculate a correlation between SLC7A5 and tumor-associated immune cells. Correlation coefficient is indicated by the color. When an asterisk appears, it indicates a statistically significant p-value based on spearman correlation analysis. *P＜0.05; **P＜0.01; ***P＜0.001 [file 12935_2024_3365_MOESM26_ESM.tif]

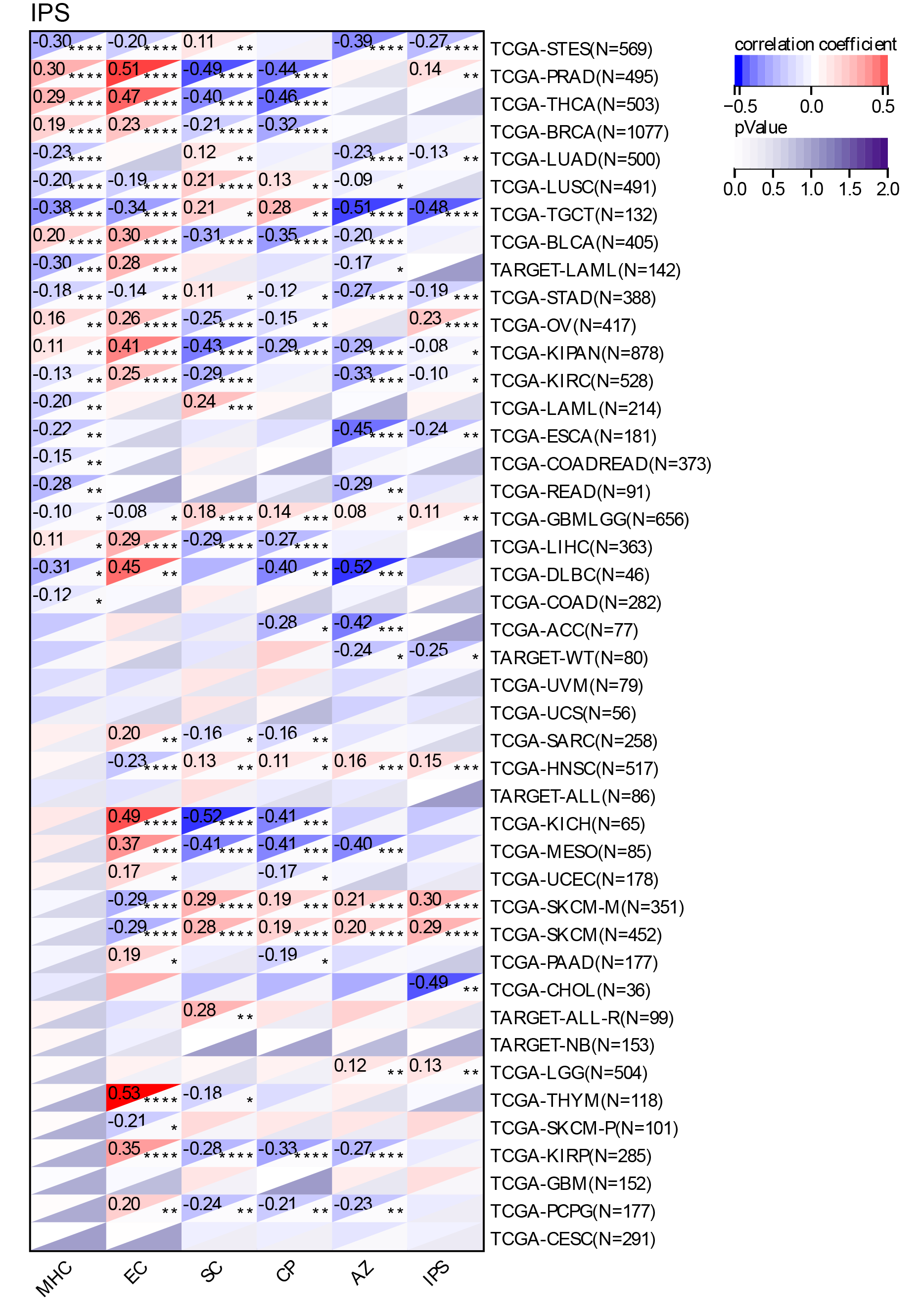

Supplement: Supplementary file 27 — Supplementary Material 27: Supplement figure 27. The IPS algorithm was used to calculate a correlation between SLC7A5 and tumor-associated immune cells. Correlation coefficient is indicated by the color. When an asterisk appears, it indicates a statistically significant p-value based on spearman correlation analysis. *P＜0.05; **P＜0.01; ***P＜0.001 [file 12935_2024_3365_MOESM27_ESM.tif]

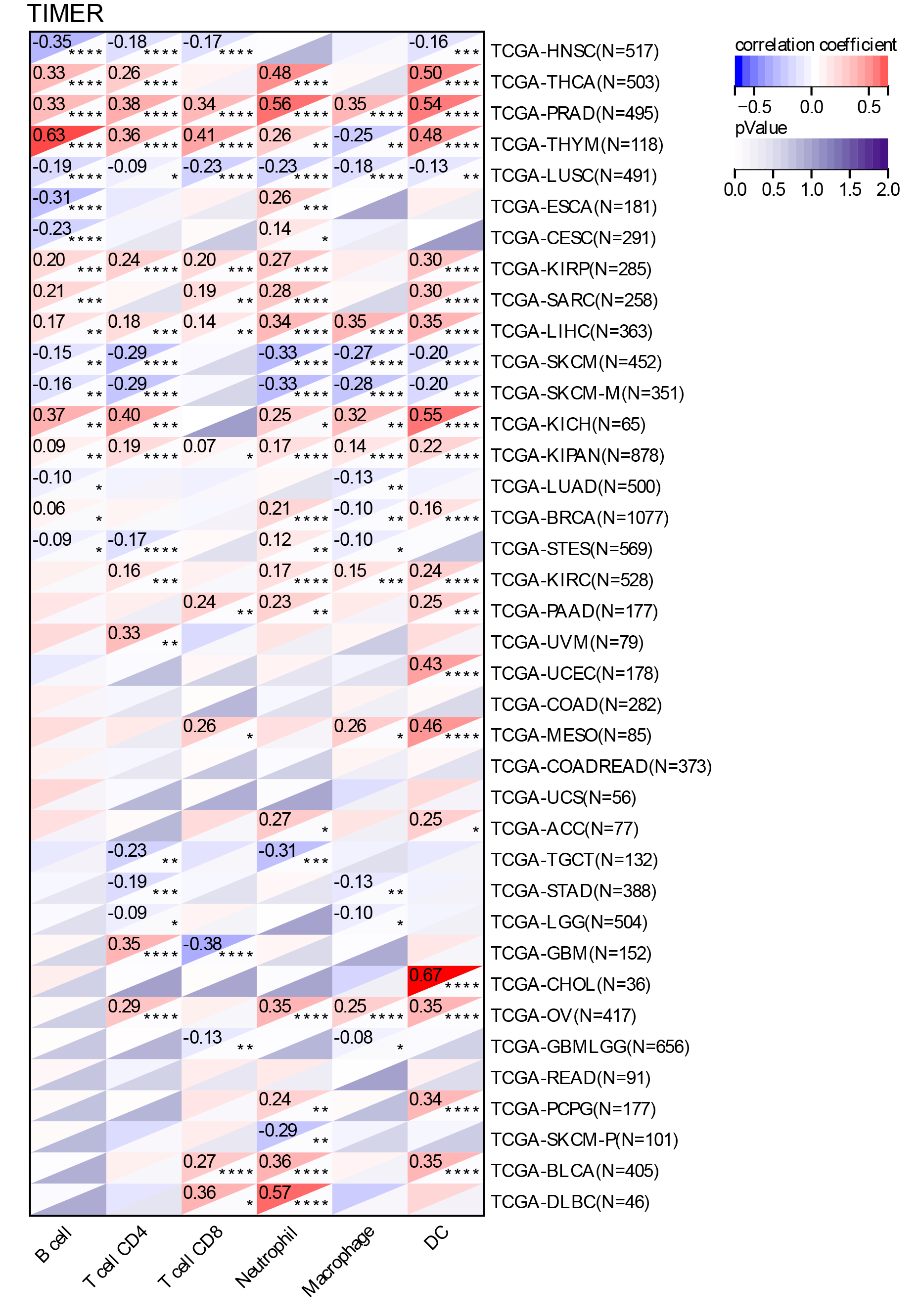

Supplement: Supplementary file 28 — Supplementary Material 28: Supplement figure 28. The TIMER algorithm was used to calculate a correlation between SLC7A5 and tumor-associated immune cells. Correlation coefficient is indicated by the color. When an asterisk appears, it indicates a statistically significant p-value based on spearman correlation analysis. *P＜0.05; **P＜0.01; ***P＜0.001 [file 12935_2024_3365_MOESM28_ESM.tif]

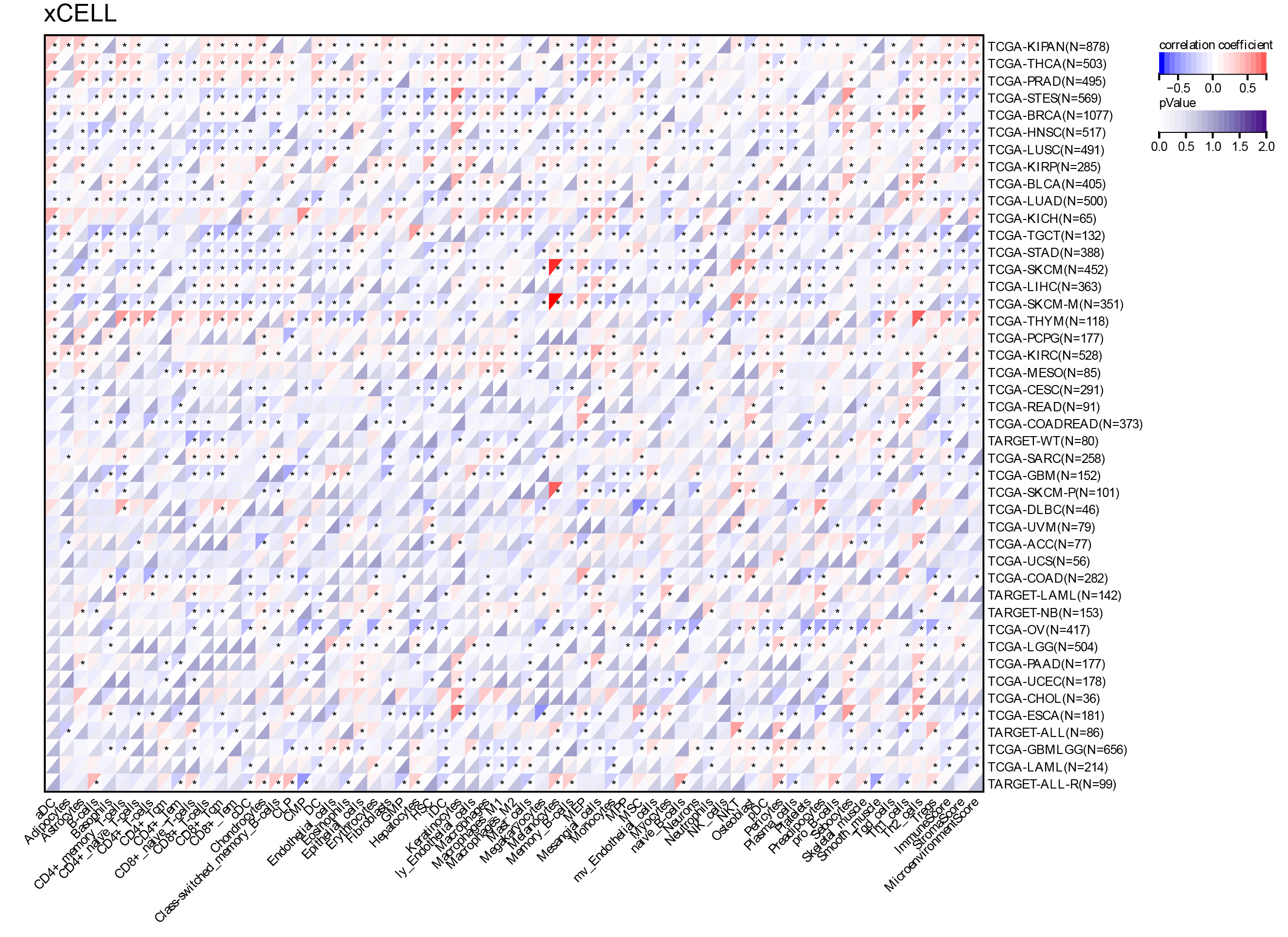

Supplement: Supplementary file 29 — Supplementary Material 29: Supplement figure 29. The xCELL algorithm was used to calculate a correlation between SLC7A5 and tumor-associated immune cells. Correlation coefficient is indicated by the color. When an asterisk appears, it indicates a statistically significant p-value based on spearman correlation analysis. *P＜0.05; **P＜0.01; ***P＜0.001 [file 12935_2024_3365_MOESM29_ESM.tif]

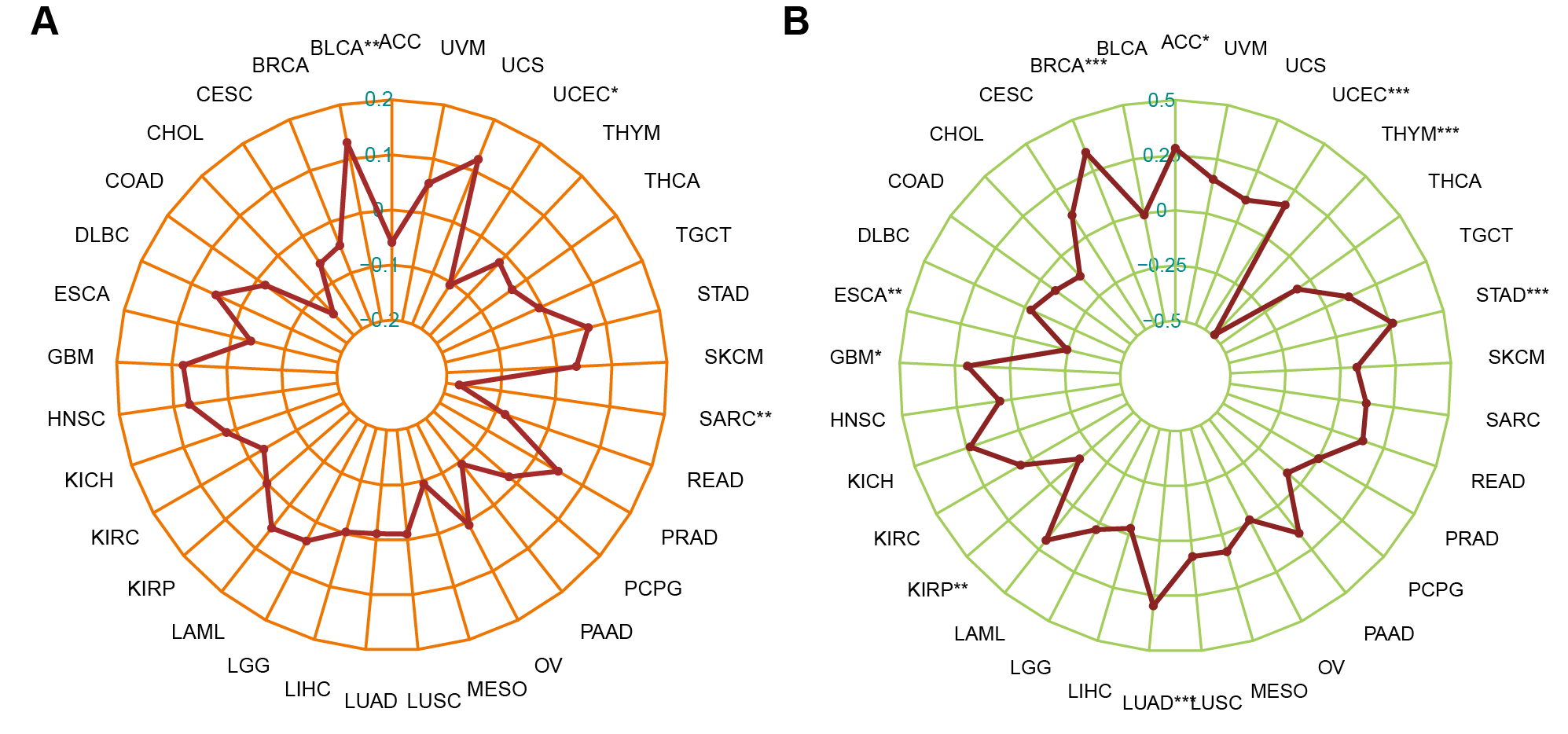

Supplement: Supplementary file 30 — Supplementary Material 30: Supplement figure 30. In pan-cancers, SLC7A5 is correlated with TMB and MSI. A. Correlation between SLC7A5 and TMB in pan-cancers. B. SLC7A5 and MSI correlation in pan-cancers. Asterisks indicate significant statistical p values calculated using spearman correlation. *P＜0.05; **P＜0.01; ***P＜0.001 [file 12935_2024_3365_MOESM30_ESM.tif]

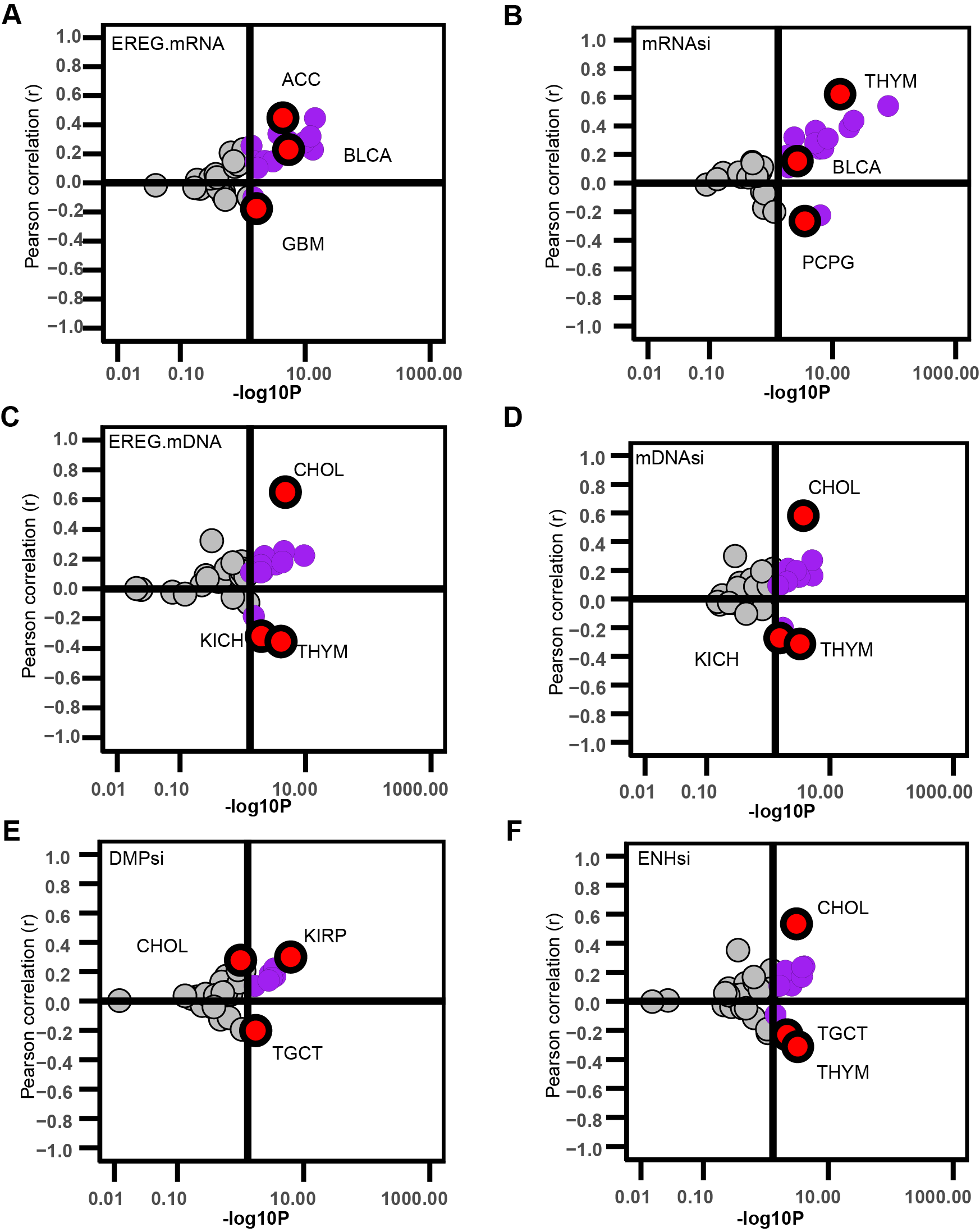

Supplement: Supplementary file 31 — Supplementary Material 31: Supplement figure 31. Correlations between SLC7A5 and cancer stemness indices across cancers [file 12935_2024_3365_MOESM31_ESM.tif]
